# Structured microalgal bioprints for enhanced photosynthetic performance and growth

**DOI:** 10.3389/fbioe.2026.1755740

**Published:** 2026-07-06

**Authors:** Swathi Murthy, Maria Mosshammer, Michael Kühl

**Affiliations:** Department of Biology, Marine Biological Section, University of Copenhagen, Helsingør, Denmark

**Keywords:** bioprinting, light transfer, mass transfer, microalgae, predictive modeling

## Abstract

Efficient cultivation of microalgae for biofuel and bioproduct applications is often limited by suboptimal light distribution, poor mass transfer of chemical species, and large land footprint requirements in conventional flat biofilm and open-pond systems. In this proof-of-concept study, we employ a closed-loop, computation-guided framework that integrates predictive modeling, 3D bioprinting, and quantitative experimentation to design and explore the influence of bioprinted microalgal geometry on photosynthetic production, where experimentally derived parameters are reintegrated into simulations to interpret performance. Specifically, we investigate a perforated slab with channels (PS) and Vgroove structures, which exhibited higher measured algal growth rates, under the present experimental conditions, compared to flat slabs of the same volume and footprint. By integrating experimentally measured photosynthetic parameters into radiative transfer and oxygen diffusion–reaction simulations, we analyze the potential contribution of geometry-induced differences in light penetration, surface area-to-volume ratio, and mass transfer dynamics that are consistent with the observed trends in algal growth and photosynthesis. These findings illustrate the potential of engineered 3D bioprinted architectures to optimize photosynthetic efficiency and growth rates, while minimizing spatial footprint, paving the way for compact, high-throughput bioreactors for sustainable algal cultivation.

## 1 Introduction

To meet the world’s growing energy demands in a sustainable manner, microalgae have emerged as highly appealing biomass feedstock for biofuel production due to their environmental compatibility, economic viability, renewability, and their ability to produce valuable byproducts (Suali and Sarbatly, 2012; Mata et al., 2010; Amer et al., 2011). Microalgae contribute to climate change mitigation by sequestering CO_2_ during photosynthesis and require substantially less land area than terrestrial crops, owing to their superior photosynthetic efficiency, which can be 10–50 times higher (Suali and Sarbatly, 2012; Mata et al., 2010; Amer et al., 2011). Moreover, biofuel production efficiency from microalgae is strongly linked to their lipid content and biomass productivity, which can reach up to 80% (dry weight basis) and 7.3 g L^-1^ day^-1^, respectively, making them attractive candidates for sustainable bioenergy systems (Suali and Sarbatly, 2012). Despite these advantages, the large-scale commercialization of algal biofuels remains limited by economic challenges, primarily associated with insufficient biomass productivity. These limitations arise from inefficient utilization of incident light energy and inadequate mass transfer of nutrients and gases within dense algal cultures (Suali and Sarbatly, 2012; Mata et al., 2010; Amer et al., 2011; Cho et al., 2019). As a result, improving cultivation strategies and reactor designs is critical for enhancing productivity and reducing production costs.

The choice of cultivation system, such as open ponds or photobioreactors, plays a central role in determining biomass productivity by controlling key environmental factors, including light availability, CO_2_ supply, oxygen removal, and nutrient transport (Suali and Sarbatly, 2012; Amer et al., 2011). Autotrophic cultivation systems, particularly flat-panel photobioreactors, offer relatively low production costs but are fundamentally constrained by poor light penetration and large spatial footprints (Ruiz et al., 2022). In contrast, heterotrophic cultivation enables much higher cell densities and volumetric productivities through the use of organic carbon sources; however, it sacrifices direct CO_2_ utilization and increases operational costs (Barros et al., 2019; Doucha and Lívanský, 2012). These trade-offs highlight the need for innovative reactor architectures that improve light harvesting and mass transfer while maintaining space efficiency.

Natural phototrophic biofilms face inherent limitations in light penetration, which restricts their photosynthetic efficiency (Bengtsson et al., 2018). Advanced photobioreactor designs that promote uniform light distribution and enhanced mass transport could overcome these limitations and substantially improve algal productivity (Suali and Sarbatly, 2012; Mata et al., 2010; Amer et al., 2011; Cho et al., 2019; Parmar et al., 2011; C et al., 2024). Inspiration for such designs can be drawn from biological systems that have evolved highly efficient light-management strategies, such as reef-building corals and giant clams, which host microalgal symbionts within intricately structured tissues that enhance photosynthesis through optimized optical and mass-transfer properties (Stambler and Dubinsky, 2005; Brodersen et al., 2014; Holt et al., 2024).

Computational and experimental studies further demonstrate that optimized reactor geometries and optical configurations can dramatically increase microalgal biomass productivity, potentially exceeding 100 g dry biomass m^-2^ day^-1^ under optimal conditions (Cho et al., 2019). For example, V-shaped bioreactor configurations have shown enhanced light trapping and dispersion, resulting in significant gains in biomass productivity, up to two fold compared to flat systems (Cho et al., 2019). Since the economic viability of algal biofuel production is tightly coupled to achievable biomass yields, predictive modeling and simulation of photophysiological performance have become essential tools for rational reactor design and scale-up (Murthy et al., 2025; Bao et al., 2023).

Computational methods originally developed in biomedical optics for simulating light propagation in biological tissues (Sassaroli and Martelli, 2012; Leino et al., 2019; Zhu and Liu, 2013; Yuan et al., 2021) can be integrated with hydrodynamic and mass-transfer models (Taylor Parkins et al., 2021; Murthy et al., 2023) to guide optimal design of algal cultivation platforms. These computational designs can be rapidly prototyped using methods such as 3D bioprinting (Murthy et al., 2025; Levato et al., 2020; Groll et al., 2016; Hospodiuk et al., 2017), a technique widely employed in tissue engineering for precise spatial patterning of cells within hydrogels (Derby, 2012; Zhang et al., 2013; Schuurman et al., 2013; Billiet et al., 2012). In comparison, the application of bioprinting to algal systems remains relatively under explored.

Several studies have explored the 3D printing and immobilization of microalgae and demonstrated that scaffold architecture plays a critical role in regulating algal viability, growth, and transport processes. Extrusion-based bioprinting approaches have been used to fabricate photosynthetically active living materials, highlighting the importance of hydrogel architecture in maintaining microalgal viability and function (Krujatz et al., 2015). Biomimetic bioprinted constructs inspired by coral–microalgae symbioses have further shown that spatial organization and light management strongly influence photosynthetic performance (Wangpraseurt et al., 2020). Other developments on microalgae-based 3D bioprinting include the development of improved hydrogel bioinks that enhance structural stability and long-term cell viability, high-resolution printing strategies that enable precise spatial control and improved light and mass transfer (Murthy et al., 2025), and the emergence of engineered living materials for applications such as carbon capture, bioremediation, and sustainable bioproduction (Tang et al., 2025). Beyond structural design, strategies such as controlled algal aggregation and the incorporation of light-scattering particles have been shown to enhance internal light distribution, leading to up to fourfold increases in photosynthetic efficiency compared to conventional biofilms (Chua et al., 2024). Recent advances combining 3D bioprinting, non-invasive imaging, and computational modeling now enable precise control and monitoring of engineered algal constructs, providing new opportunities to optimize photosynthetic performance and biomass productivity (Murthy et al., 2025).

While these prior studies establish that scaffold design can influence algal performance, they rely primarily on empirical observations. In contrast, the present work focuses on examining geometry-dependent influences on algal growth and photosynthetic performance under controlled conditions. We couple experimental measurements with light and mass transfer simulations to provide mechanistic interpretation. We present a proof-of-concept study that integrates computational simulations, 3D bioprinting, and metabolic measurements to design and investigate structured algal geometries exhibiting higher measured growth rates, under the present experimental conditions, relative to conventional flat biofilms (Fanesi et al., 2019) and open-pond cultivation systems (Suali and Sarbatly, 2012; Mata et al., 2010). Our objective is to engineer space-efficient architectures that improve light distribution and mass transfer characteristics, enabling high-density algal growth within a minimal land footprint.

We employ a computation-driven design strategy, in which candidate geometries are first evaluated using predictive simulations of light distribution and mass transfer under defined algal concentrations and material properties. The simulation-informed designs are then fabricated via 3D bioprinting and evaluated experimentally to compare experimental observations with model predictions. Importantly, experimentally derived parameters, including biomass concentration, optical properties, and photosynthetic O_2_ production rates, are subsequently incorporated into post-experimental simulations to interpret the observed growth trends and elucidate the underlying physical and physiological mechanisms. By integrating mechanistic modeling with targeted experimentation in a closed-loop framework, this approach enables systematic investigation and iterative refinement of algal cultivation architectures, while minimizing reliance on empirical trial-and-error. The presented fabrication–simulation pipeline is broadly applicable to studying other structure–function relationships inspired by natural systems (Parker and Townley, 2007; Burresi et al., 2014), including symbiosis (Dimijian, 2000), and microbe-host interactions (Lupp, 2007; Medzhitov, 2007; Turnbaugh et al., 2007), thereby advancing predictive design strategies for sustainable biofuel production and enabling deeper understanding of the underlying biological mechanisms.

## 2 Methods

Gelatin from porcine skin (gel strength 300, Type A), methacryl anhydride (containing 2,000 ppm topanol A as inhibitor, 94%), NaHCO_3_, and poly(1-vinylpyrrolidone-co-styrene) (38% emulsion in H_2_O, <0.5 μm particle size) were purchased from Sigma Aldrich (sigmaaldrich.com) and used without further modification. The tangential flow filtration (TFF) system was generously provided by PALL (pall.com/), and the MinimateTM TFF capsule with a 10K omega membrane was purchased from PALL. TAP medium was prepared in MilliQ water, according to the recipe: https://utex.org/products/tap-medium?variant=30991736897626#recipe. 0.25% solution of Trypsin-EDTA was purchased from Sigma Aldrich (sigmaaldrich.com). Irgacure 2959 was purchased from BASF (IC2959; BASF, cat. no. 029891301PS04) and Lithium phenyl-2,4,6-trimethylbenzoylphosphinate from Sigma Aldrich (LAP; Sigma Aldrich).

### 2.1 GelMA synthesis

Functionalized gelatin methacryloyl (GelMA) based hydrogels were synthesized according to literature (Loessner et al., 2016). Briefly, 10 g of gelatin was swelled for 30 min at room temperature and fully dissolved at 50 °C in a water bath under stirring in 100 mL MilliQ water. 4 g of methacrylic anhydride was added dropwise to the gelatin solution under vigorous stirring. All the subsequent steps were conducted at low light levels or darkness. The solution was stirred for 1h and centrifuged at 3,500 rpm for 3 min at room temperature. The pellet was discarded and the clear supernatant was diluted with 2 volumes of deionized water heated to 40 °C. This was followed by dialysis at 40 °C for 5 days using a Minimate™ TFF capsule (10 kDa cut-off). The pH was adjusted to pH 7.4 using NaHCO_3_ (1M), and the solution was filter-sterilized using a vacuum filtration unit with a 0.2 µm filter (PES membrane). The GelMA solution was transferred to 50 mL vials with vented screw caps, snap frozen and lyophilized at −55 °C in a freeze dryer (CoolSafe 55–4 Pro, Ninolab) for 7 days. The lyophilized GelMA was stored at −20 °C prior to usage.

### 2.2 Algal culture

A culture of the green alga *Chlorella sorokiniana* UTEX1230 was maintained in culture tubes with TAP medium (see above) under constant shaking and under a defined photon irradiance from white LED’s (10 μmol photons m^−2^ s^−1^; 400–700 nm) in a temperature-regulated culture cabinet (ALGAETRON, Photon System Instruments) at 24 °C.

### 2.3 Bio-ink preparation

A photo initiator stock solution was prepared by dissolving Lithium phenyl-2,4,6-trimethylbenzoylphosphinate (LAP; Sigma Aldrich, CAS Number 85073-19-4) in 10 mL TAP medium at 70 °C under constant stirring, where after the solution was allowed to cool to room temperature (Loessner et al., 2016). 8% (w/v) GelMA ink was prepared, by soaking GelMA foam in TAP medium with added photo-initiator stock solution (to final concentration, w/v of 0.25%), overnight in an refrigerator. This was followed by dissolving the GelMA-containing TAP solution in an incubator at 37 °C and stirring at 300 rpm, until the mixture turned clear (Loessner et al., 2016). The pH of the solution was adjusted to pH 8.1 by adding a strong base (1M NaOH). The GelMA ink was finally heat treated at 80 °C for 15 min as a disinfection step. The ink was cooled to <30 °C, before adding the microalgal cells. The algal cells were spun down from the stock solution at 9,000 rpm for 60 s. The supernatant was discarded, and the algal cells were added at a concentration of 19 ± 1.2 x 10^6^ cells mL^-1^ bioink to obtain a relatively low biomass load. At ∼26 °C, the hydrogel exhibited low viscosity, allowing the hydrogel–algal cell mixture to be pipetted up and down several times to achieve homogeneous cell distribution within the bioink. The microalgal culture used in the study was non-axenic; therefore, the bio-ink contained associated bacteria.

### 2.4 Bioprinting

Three different construct geometries with similar volume and food print, i.e., a construct with V-grooves (Cho et al., 2019) (30° vertex angle, 4.7 mm height, volume 123.28 mm^3^, footprint 52.34 mm^2^), a perforated slab construct (volume 175.64 mm^3^, footprint 72.25 mm^2^) and a corresponding simple slab structure, were designed in CAD software (Autodesk Fusion 360. ink), followed by slicing and g-code creation in PrusaSlicer (PrusaSlicer.ink). The g-code was uploaded and printed on a commercial 3D bioprinter (Bio-X, CellInk Lifesciences), which utilizes an extrusion-based printing technique. The printing was carried out with an infill density set to 60% using a 3D honeycomb pattern. The print speed and extrusion pressure were set to 2 mm s^-1^ and 25 kPa, respectively. The temperature of the bio-ink was set to 24 °C, to obtain a desired viscosity for printing. The constructs were printed, on a PET foil substrate maintained at 10 °C and were allowed to stand for 5 min (after printing the entire construct) to facilitate physical cross-linking, before curing with 405 nm light, at intensity of 3 mW cm^-2^ for 30 s. The prints were stored in sterile Petri dishes with TAP medium sealed off at the edges with parafilm. The printing and handling were carried out in a sterile environment, either in the bioprinter (fitted with UV-C germicidal lamps and a HEPA H14 dual-filter system) or in a laminar flow bench. The prints were stored in an incubation chamber under constant photon irradiance (PAR; 400–700 nm) of 10 μmol photons m^−2^ s^−1^ from white LED’s (AlgaeTron AG 230) between day 0 and day 1, and under 60 μmol photons m^−2^ s^−1^ between day 1 and day 4. The printing procedure described in our previous work (Murthy et al., 2025) was followed to ensure that fabrication and incubation did not introduce contamination. Acellular printed constructs incubated under identical conditions remained stable for several months, indicating that the fabrication process itself did not introduce unintended contamination.

### 2.5 Biomass quantification via cell counts

The cell density at the beginning of the experiments (day 0) was estimated by measuring the cell concentration in the algal stock solution (as described below), before adding it to the bio-ink. In our previous work using the same printing protocol (Murthy et al., 2025), we demonstrated that printing with LAP photoinitiator (405 nm curing) had minimal effect on cell viability, whereas printing with Irgacure (365 nm curing), caused visible bleaching of cells at construct edges. This was confirmed both visually and through variable chlorophyll fluorescence measurements. Hence a geometry-specific post-print Day 0 cell count was not performed in this study. We note that potential effects of the printing process, such as the pre-cure period, substrate temperature, and depth-dependent curing, on initial cell retention or spatial redistribution within different geometries cannot be fully decoupled from subsequent growth dynamics. As a result, the reported cell densities might not fully decouple geometry-induced effects on initial (day 0) cell distribution and therefore primarily reflect comparative growth outcomes under identical fabrication and incubation conditions, rather than absolute differences in initial cell loading. However, cell viability after printing was assessed using variable chlorophyll fluorescence imaging (VCFI), as described in Section 2.7. Measurements were performed 12 h after printing (designated as day 1). During the period between day 0 and day 1, all samples were maintained under low illumination (10 μmol photons m^-2^ s^-1^) to minimize biomass growth.

The cell concentration in the bio-prints on day 4 was determined by removing the prints from the growth medium and dissolving them in 1,000 µL trypsin solution (Liu et al., 2016) (0.25% Trypsin/EDTA) at 37 °C for 1 h. For cell counting, the algal solution (stock culture samples or dissolved prints in trypsin) was diluted 3,000 to 4,000 times in TAP medium, depending on the algal cell concentration. Then 1 mL of the diluted algal solution was poured into a Sedgewick rafter counting chamber, which subdivides 1 mL into (1,000) 1 µL volume fractions. The number of algal cells in each 1 µL volume fraction was manually counted under an optical microscope. At least 15 to 20 technical replicates/volume fractions were counted for each sample and averaged. Mean ± standard deviation values are reported for each sample. To ensure consistent cell recovery across samples, printed constructs were allowed to fully dissolve in the trypsin solution, and the resulting suspensions were thoroughly vortexed prior to sampling for cell counting. While this procedure was applied uniformly to all geometries, we note that the measured cell counts reflect comparative outcomes rather than absolute recovery efficiencies. However, care was taken to perform cell counts immediately after dissolution to ensure that all released cells were accounted for, including any potentially non-viable cells that may have resulted from the dissolution process. We note that in this proof-of-concept study, the number of biological replicates for growth comparisons is effectively n = 1 per geometry. Consequently, reproducibility, print-to-print variability, and bio-ink–to–bio-ink variability cannot be quantitatively assessed in the current dataset. This limits the strength of comparative claims. Accordingly, the reported growth enhancements should be interpreted as comparative trends between geometries fabricated and cultured under identical conditions, providing a proof-of-concept for the simulation-informed framework to link construct architecture with underlying physical and physiological processes.

### 2.6 Optical coherence tomography (OCT) measurements and analysis

A spectral domain OCT system (Ganymed II; Thorlabs GmbH, Dachau, Germany) equipped with an objective lens with an effective focal length of 18 mm and a working distance of 7.5 mm (LSM02-BB; Thorlabs GmbH, Dachau, Germany) was used for OCT imaging (Wangpraseurt et al., 2017). The system is equipped with a 930 nm light source, yielding a maximal axial and lateral resolution in water of 5.8 μm and 8 μm, respectively. Two-dimensional OCT B-scans were acquired at a fixed pixel size of 584 x 1,024. The actual field of view was variable in y but fixed in z (=2.2 mm). The OCT system was optimized to yield highest signal at a fixed distance, in the upper 1/3rd of the image (Wangpraseurt et al., 2017). OCT imaging was performed on bio-printed constructs fully immersed in TAP medium in a Petri-dish. System calibration and optical parameter extraction were performed according to previously published procedures (Murthy et al., 2025; Wangpraseurt et al., 2018; Samatham and Jacques, 2009; Samatham et al., 2008). OCT has been widely employed as a non-invasive quantitative imaging tool for the structural characterization and longitudinal monitoring of biological tissues, engineered living materials, and biofabricated constructs, enabling the assessment of internal morphology and structural changes without disturbing the sample (Bao et al., 2023; Tashman et al., 2022).

### 2.7 Variable chlorophyll fluorescence imaging

The photosynthetic performance of the microalgae in the bioprinted constructs was measured via a pulse-amplitude-modulated, variable chlorophyll fluorescence imaging system (I-PAM/GFP, Walz GmbH, Effeltrich, Germany) using the pulse-saturation technique (Schreiber, 2004b; Ralph et al., 2005). The system utilizes blue (470 nm) LED light for weak (<1 μmol photons m^−2^ s^−1^) modulated measuring light pulses, strong (0.8 s at >2,500 μmol photons m^−2^ s^−1^) saturating light pulses, and defined levels of blue actinic irradiance, as measured with a calibrated photon irradiance meter at the level of the bioprinted constructs (ULM, Walz, Effeltrich, Germany). Fluorescence parameters were spatially averaged over the entire surface area of each bioprinted construct.

From measurements of the minimum fluorescence yield, *F*_*0*_, and the maximum fluorescence yield, *F*_*m*_, in dark acclimated samples, as recorded before and during a saturation pulse, respectively, we calculated the maximum quantum yield of PSII as *F*_*v*_ */F*_*m*_ *= (F*_*m*_ *-F*_*0*_ *)/F*_*m*_. This parameter is frequently used to indicate the health and photosynthetic capacity of photosynthetic organisms (Schuurmans et al., 2015). The effective PS II quantum yield in light-exposed samples was calculated as *YII = (F* ^*’*^_*m*_ *-F)/F* ^*’*^_*m*_, where *F’*_*m*_ is the maximal fluorescence yield of light acclimated samples (under the saturating light pulse) and *F* is the fluorescence yield under ambient actinic light conditions (Schreiber, 2004b; Ralph and Gademann, 2005).

From YII measurements after a 10 s exposure at each light level, covering over a range of increasing actinic photon irradiance levels of photosynthetic active radiation (PAR; 400–700 nm), we calculated a so-called rapid light curve (RLC) (Ralph and Gademann, 2005). The RLC quantifies how the relative electron transport rate (rETR = YII x E_d_) via PSII changes with increasing photon irradiance, E_d_, and can be used as a qualitative measure of the photo-physiological acclimation state of the micro-algae embedded in the prints (Ralph and Gademann, 2005).

### 2.8 Gas exchange measurements

The net O_2_ exchange between bioprinted constructs and their surrounding medium was measured with samples kept in a customized, gas-tight glass chamber (10 mL volume) with a flat, glass coverslip as cover (Murthy et al., 2025). The edges of the chamber were painted black, to avoid light guiding and scattering effects. The construct was placed on a small, flat holder inside the chamber above a stirring bar, ensuring constant flow and efficient mass transfer between construct and the surrounding water. An optical O_2_ sensor spot with an optical isolation (PyroScience GmbH) was mounted inside the chamber and could be read out via an optical fiber attached to the chamber at one end and to a fiber-optic O_2_ meter (FireStingO_2_; PyroScience GmbH) at the other end. Calibration of the sensor spots were done according to the manufacturer, and O_2_ concentrations inside the experimental chamber were logged every 10 s enabling a quantification of the net O_2_ exchange between the bioprinted constructs and the surrounding water in the closed chamber.

The net O_2_ production, J, per constructs, where positive values indicate net production and negative values indicate net consumption of O_2_, was calculated, according to Equation 1 from the measured linear change in O_2_ concentration in the chamber over time, dC/dt, corrected for the water volume surrounding the print (V) and the printed construct volume (V_construct_):

$$J=\frac{dC/dt*V}{Vconstruct}$$ (1)

The O_2_ flux was normalized to print volume since the structured print and its corresponding slab had identical volumes, enabling direct comparison of their rates. For recording of O_2_ dynamics, the bioprinted constructs were kept in darkness for 10 min prior to switching on the light source, i.e., a fiber-optic tungsten-halogen lamp (K2500 LCD, Schott GmbH). The incident photon irradiance of photosynthetically active radiation (PAR; 400–700 nm) at the level of the construct surface in the chamber was measured with a calibrated spectroradiometer (BTS 256, Gigahertz Optics GmbH) for different lamp settings. The bioprints were then exposed to increasing photon irradiance (100, 200, 270, 430 and 530 µmol photons m^-2^ s^-1^; 400–700 nm) with intermittent dark periods for about 18 min each. Measurements were logged continuously every 10 s. The flux measurements were used to determine the net production (NP) and dark respiration (DR) rates after a period of illumination, where after the gross photosynthesis rate was estimated as (NP + DR). The different bioprinted constructs were measured simultaneously in separate vials. The net production of O_2_ as a function of incident photon irradiance was fitted to an exponential function according to Spilling et al. (Spilling et al., 2010) and the estimated gross photosynthesis according to Webb et al. (1974)

### 2.9 Approximation of optical properties for simulation

The optical properties of bio-prints used for simulating the light distribution (see 2.11) were approximated from either the cell count or the OCT data (Section 2.6). The algal cell count was used to calculate the absorption coefficient, µ_a,_ and the scattering coefficient, µ_s_, based on an earlier experimental study of the green microalga *Chlorella vulgaris* (Kandilian et al., 2016). To account for the algal growth dynamics, calibrated OCT images (Figure 3) were used to extract the scattering coefficient, µ_s,_ and the scattering anisotropy factor, g, for samples on day 4 (summarized in Supplementary Table S1). It was assumed that the extracted µ_s_ at 930 nm would be similar to the value at 636 nm (used for simulations).

### 2.10 Calculation of net-photosynthesis quantum efficiency

The net-QE for all the samples on day 4 was calculated, according to Equation 2, under an incident photon irradiance of 430 µmol photons m^-2^ s^-1^ (400–700 nm), from the gas exchange data shown in Figures 3b,d. At the selected photon irradiance, all samples exhibited net O_2_ production. The following equation was used to calculate the net-QE (Al-Najjar et al., 2010):

$$net\_QE=\frac{net\_PS}{\mu_{a}\;I_{p}\;}$$ (2)

where net_*QE* is the quantum efficiency (net production) of algal photosynthesis, net_PS is net production of O_2_ obtained from respirometery data (Figures 3b,d), µ_a_ is the absorption coefficient of the bioprint obtained from cell count (Supplementary Figure S7), *I*_*p*_ is the photon scalar irradiance.

### 2.11 Monte Carlo simulation of radiative transfer in bioprinted constructs

Monte Carlo (MC) modelling was used to calculate the radiative transfer in the bioprinted constructs (Wang et al., 1995) using the free finite-element MC simulation software ValoMC (Leino et al., 2019), employing the algorithm described by Prahl *et al.* (Prahl et al., 1989), in combination with COMSOL Multiphysics (v5.6, COMSOL Inc., Burlington, MA), as described in our previous study (Murthy et al., 2025). Briefly, the discretized three-dimensional geometry (created in COMSOL, minimum tetrahedral element size 3 µm) was loaded into ValoMC. A set of material properties was assigned to each of the model domains (summarized in Supplementary Table S1), consisting of *μ*_*a*_, *μ*_*s*_, *g* (using the Henyey-Greenstein approximation for the scattering phase function), and the refractive index *n* (1.33*)*. A ‘direct’ light source at 636 nm, was assigned over the entire top boundary (as shown in Supplementary Figure S2) with a total power of 1 W. For each simulation, 10^8^ photon packets were launched. The output scalar irradiance from the MC simulation was normalized to the incident photon irradiance. The normalized light field was imported into COMSOL and mapped over the 3D bio-print model.

The photon scalar irradiance *I*_*p*_ was calculated from the normalized scalar irradiance (*I*_*s*_), as shown in Equation 3.

$$I_{p}=\frac{I_{s}\;I_{id}}{E_{p}\;N_{A}}$$ (3)

where *I*_*id*_ is the incident downwelling irradiance, *N*_*A*_ the Avogadro’s number, and *E*_*p*_ the energy of a photon at 636 nm, ( λ ), calculated as *h*c/ λ , h is Planck’s constant, and c is the velocity of light in vacuum.

### 2.12 Mass transfer simulation

Mass transfer simulations were carried out in COMSOL Multiphysics (v5.6, COMSOL Inc., Burlington, MA).

*Fluid flow*: Stationary and incompressible Navier-Stokes equations for laminar flow were used to simulate the water flow over the bio-print in a flow chamber (Supplementary Figure S1), as described in previous work (Murthy et al., 2025). A constant water density and dynamic viscosity at 25 °C was used. A fully developed laminar flow with average velocity of 5 mm s^-1^ was assigned to the inlet. The transport of the photosynthetically produced O_2_ in the bioprint, was supported by the water velocity profile calculated over the bio-print (described under oxygen transport and reactions). A mesh-independent study (Supplementary Figure S2) showed that the chosen mesh size, minimum element size of 4 µm for light and 82 µm for O_2_ simulation, is sufficient.

*Oxygen transport and reactions*: The dissolved oxygen concentration (*c*_*O2*_), was calculated from diffusion-reaction equations in the bio-print and from diffusion-convection equation in the water column, as explained in our previous work (Murthy et al., 2025). The net rate of O_2_ produced by photosynthesis in the bio-print was calculated

$$RO_{2}=\left(net\_QE\right)\;\mu_{a}I_{p}$$ (4)

where net_*QE* is the quantum efficiency (net production) of algal photosynthesis (assumed to be 0.005 for day 0 or calculated from respirometry measurements on day 4), µ_a_ is the absorption coefficient of the bioprint, *I*_*p*_ is the photon scalar irradiance.

## 3 Results and discussion

**FIGURE 1:**
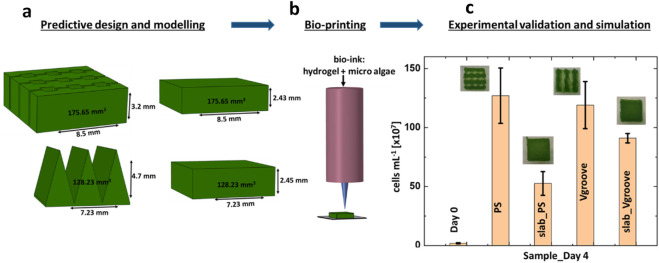
Simulation-guided design, fabrication, and evaluation of structured algal constructs. **(a)** Predictive designs of algal bioprint geometry optimized using simulations of light propagation and mass transfer, including a perforated slab (PS; holes interconnected by channels), a V-groove structure with a 30° opening angle, and corresponding flat slab controls with identical volume and footprint; **(b)** Schematic sketch of bioprinting of the designs in GelMA hydrogel mixed with microalgae; **(c)** Algal cell counts in the different samples on day 4 after printing. Insets show photographs of the constructs submerged in growth medium on day 4. Number of biological replicates per geometry was 1 (n = 1).

Using predictive modeling as the primary design tool in this proof-of-concept study, we illustrate how the integration of computational simulations, 3D bioprinting, and algal metabolism measurements enables the systematic exploration of geometries associated with higher measured algal growth rates, under the present experimental conditions, relative to conventional flat structures. Light propagation and mass transfer were first simulated assuming a defined algal biomass concentration and spatial distribution, from which the effective optical properties (Kandilian et al., 2016) and photosynthesis rates of the algal medium were derived. Based on these simulations, two representative geometries, a perforated slab (PS) and a V-groove structure with a 30° opening angle, were selected for experimental implementation (Figure 1a). These designs were computationally identified as promising candidates due to their predicted ability to improve light penetration and/or mass transfer, as compared to a flat slab with identical volume and footprint (Supplementary Figure S3).

The simulation-informed geometries were subsequently fabricated via 3D bioprinting in GelMA hydrogel containing microalgae (Figure 1b; Supplementary Figure S4) and incubated in growth medium under constant illumination with an incident photon irradiance (400–700 nm) of 60 μmol photons m^-2^ s^-1^ for up to 4 days. Although the print fidelity and contamination control during fabrication were previously established using the same printing protocol in our prior work (Murthy et al., 2025), the incubation period in this study was limited to 4 days due to rapid algal growth and to prevent structural deformation or disintegration. This enabled descriptive comparison of growth trends and physiological responses across samples under identical conditions, rather than statistical evaluation of performance differences, as only a single construct per geometry was examined (n = 1). Optical and metabolic measurements were then performed to experimentally characterize sample responses and to extract key physiological parameters. These parameters were incorporated into post-experimental simulations of light transport and O_2_ mass transfer to interpret the observed growth trends and underlying mechanisms.

Cell count measurements obtained on day 4 (Figure 1c) revealed that both structured geometries supported higher algal growth than their corresponding flat slab controls, under the present experimental conditions. Post-experimental simulations incorporating experimentally measured material and physiological parameters further provided mechanistic context for these observations and offered insight into potential contributors to the observed trends. To distinguish the relative influence of light distribution and mass transfer, we compared the simulated changes in internal scalar irradiance and O_2_ transport for each geometry. The PS geometry maintained an average internal light field similar to that of the flat slab (∼95–96% of slab values) but exhibited enhanced exchange with the surrounding medium due to its 2.4–2.5-fold higher surface-area-to-volume ratio. In contrast, the V-groove geometry showed a marked increase in internal light availability (∼40% higher than the corresponding slab on day 4) together with a more moderate increase in surface-area-to-volume ratio (∼2-fold). These results suggest that mass-transfer enhancement may be the dominant contributor to the PS growth advantage, whereas improved light distribution appears to play a larger role in the V-groove geometry, although both processes likely contribute to the observed trends.

Specifically, the simulations indicate that improvements in both light distribution and mass transfer independently contribute to increased biomass accumulation. Structure geometry modulates the local light environment within the constructs, thereby influencing the spatial organization of algal clusters and overall photosynthetic efficiency (Figure 3; Supplementary Figure S5). By day 4, PS and V-groove constructs supported, on average, 2.4- and 1.3-fold higher algal biomass, respectively, compared to their corresponding flat slabs (Figure 1c). Analysis of light propagation and mass transfer simulations incorporating experimentally measured parameters (Supplementary Figure S6) suggests that the enhanced growth observed in the PS geometry may be associated with improved light penetration in combination with enhanced mass transfer, resulting from a 2.5-fold higher surface area versus volume, relative to the slab. The total light field (as indicated by simulations, Supplementary Figure S6) in PS remains comparable to that of a flat slab on day 0 and day 4 (∼95% as that of the slab). This combination may help maintain favorable conditions for autotrophic growth throughout cultivation. In addition, the improved mass transfer may facilitate supplementary heterotrophic metabolism, which has been shown to support higher cell densities and growth rates under light-limiting conditions (Barros et al., 2019; Doucha and Lívanský, 2012). Together, these observations are consistent with potential contributions from both autotrophic and heterotrophic processes to the observed biomass accumulation. However, direct partitioning of these metabolic pathways was not performed in the present study.

In contrast, the growth advantage observed for the V-groove geometry may be associated primarily with enhanced light availability and photosynthetic activity, resulting from improved light penetration relative to the flat slab, especially, as biomass accumulates over time. Notably, simulations indicate that by day 4 the V-groove structure maintains approximately 40% higher internal light availability compared to the slab (Supplementary Figure S6), in addition to exhibiting enhanced mass transfer. Together, these factors are consistent with sustained autotrophic growth, while also permitting complementary heterotrophic metabolism, leading to moderate but consistent biomass increases relative to flat controls under the tested conditions. Compared to the PS geometry, the V-groove exhibits a lower surface-area-to-volume ratio (PS: 2.4; V-groove: 2, Supplementary Figure S6), which may limit the extent of mass transfer enhancement and is consistent with the more moderate biomass increase observed in the V-groove relative to flat controls, under tested conditions.

### 3.1 Variable chlorophyll fluorescence imaging

Variable chlorophyll fluorescence imaging (VCFI) was used as a non-invasive tool to monitor algal photophysiology within the bioprinted constructs with minimal sample perturbation (Murthy et al., 2025; Schreiber, 2004b; Ralph et al., 2005; Ralph and Gademann, 2005). Measurements were performed on day 1 and day 4 after bioprinting to assess algal health, photosynthetic performance, and acclimation dynamics, as well as to estimate the irradiance levels at which photosynthetic saturation occurs.

**FIGURE 2:**
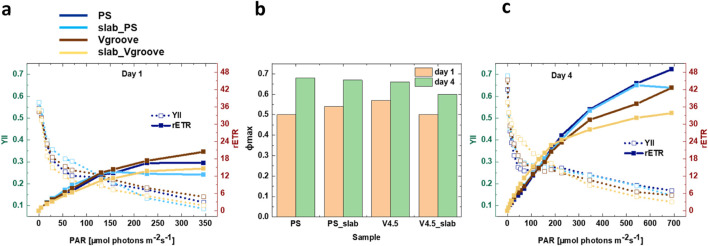
Variable chlorophyll fluorescence imaging (VCFI) assessment of photosynthetic performance in bioprinted algal constructs. **(a)** Effective PSII quantum yield, Y(II) and the corresponding relative PSII electron transport rate, rETR, integrated over the entire construct surface and plotted as a function of incident photon irradiance (400–700 nm) on day 1 after bioprinting. **(b)** Maximum PSII quantum yield (F_v_/F_m_) measured under dark-acclimated conditions on day 1 and day 4 for all constructs, indicating photosystem II integrity and photophysiological health (representative fluorescence images shown in Supplementary Figure S9). **(c)** Y(II) and rETR as a function of photon irradiance on day 4, illustrating changes in photosynthetic performance and light saturation behavior following acclimation after bioprinting. PS: perforated slab; slab_PS: flat slab control corresponding to the PS geometry; slab_V-groove: flat slab control corresponding to the V-groove geometry.

Figures 2a,c present the effective quantum yield of photosystem II, Y(II), and the corresponding relative electron transport rate (rETR), averaged over the entire construct surface, as a function of photon irradiance (400–700 nm in μmol photons m^-2^ s^-1^) on day 1 and day 4, respectively. Figure 2b summarizes the maximum quantum yield of PSII (F_v_/F_m_) measured under dark-acclimated conditions on both days, with representative fluorescence images provided in Supplementary Figure S9. On day 1 (12 h after printing), all constructs exhibited F_v_/F_m_ values of approximately 0.50–0.55. These values indicate moderate to good photosynthetic efficiency and overall physiological health of the microalgae, with only minimal transient stress following bioprinting, irrespective of print geometry. By day 4, F_v_/F_m_ values increased to approximately 0.60 – 0.70 across all samples, reflecting improved PSII functionality and recovery of photosynthetic capacity over time.

Analysis of the rETR vs. photon irradiance curves further revealed a shift in the onset of photosynthetic saturation from ∼125 μmol photons m^-2^ s^-1^ on day 1 to ∼200 μmol photons m^-2^ s^-1^ on day 4, where saturation was identified by the inflection or plateau in rETR. This upward shift is consistent with physiological acclimation of the algal photosynthetic apparatus following bioprinting, likely driven by recovery of PSII efficiency and enhanced light utilization capacity, which may enable the cells to tolerate and exploit higher irradiance levels (Schreiber, 2004a). Collectively, these measurements indicate that the microalgae remained physiologically viable throughout the experimental period and their capacity to tolerate and utilize higher irradiance levels increased as cultivation progressed.

### 3.2 Optical coherence tomography imaging and extraction of optical properties

Optical coherence tomography (OCT) was employed as a non-invasive, depth-resolved imaging technique to quantitatively characterize the spatial organization and growth dynamics of algal biomass within the bioprinted constructs. OCT is a non-invasive interferometric imaging technique that captures backscattered photons from internal microstructures and interfaces with refractive index (n) mismatches (Huang et al., 1991). Here, OCT detects backscattered photons arising from refractive index mismatches between algal cells, hydrogel, and surrounding medium, thereby providing direct information on internal microstructure and optical scattering properties.

**FIGURE 3:**
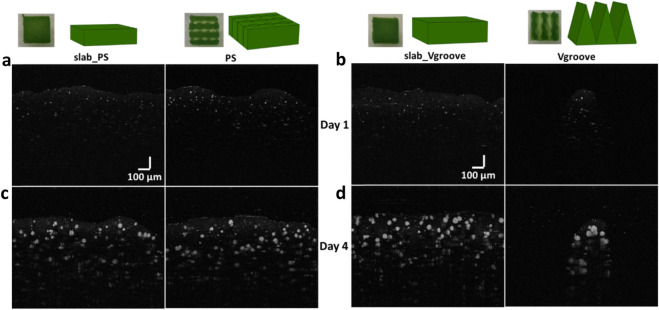
Optical coherence tomography (OCT) images of algal biomass in bioprinted constructs. OCT cross-sectional images of **(a)** flat slab control corresponding to the perforated slab geometry (slab_PS) and perforated slab (PS) on day 1; **(b)** flat slab control corresponding to the V-groove geometry (slab_V-groove) and V-groove on day 1; **(c)** slab_PS and PS on Day 4; **(d)** slab_Vgroove and Vgroove on Day 4. Inserts on top show 3D sketches and photographs of the corresponding samples. PS: perforated slab; slab_PS: flat slab control corresponding to the PS geometry; slab_V-groove: flat slab control corresponding to the V-groove geometry.

OCT imaging was performed on day 1 and day 4 after bioprinting to capture the temporal evolution of algal aggregation in both structured and flat control geometries (Figures 3a–d; see 3D renderings in Supplementary Figure S5). On day 1 (Figures 3a,b), algal clusters were relatively small, sparsely distributed, and producing weak scattering across all constructs consistent with early cell aggregation following encapsulation. The distribution of cell aggregation appeared similar across all geometries in the OCT data, as observed visually and quantitatively through optical properties extraction. This is consistent with a comparable initial cell loading and spatial distribution following the printing and curing process across the different geometries. In contrast, by day 4 (Figures 3c,d), OCT images revealed substantial increases in cluster size and density, resulting in more highly scattering biomass aggregates. These experimental observations indicate increased cell density and evolving internal optical micro environments as growth proceeded.

To quantitatively interpret these observations, the depth-resolved OCT signal intensity was analyzed using a theoretical inverse Monte Carlo framework to extract the scattering coefficient (μ_s_) and scattering anisotropy factor (g), which describe the magnitude and angular distribution of light scattering within the constructs (Levitz et al., 2010). The extracted scattering properties (summarized in Supplementary Table S1) increased markedly from day 1 to day 4, consistent with the formation of denser algal clusters and enhanced scattering. From a physical perspective, increased scattering alters photon path length, spatial light distribution, and local irradiance, thereby potentially influencing photosynthetic performance.

These experimentally derived optical properties were subsequently incorporated into light transport simulations to compute internal photon distributions, which then served as inputs for oxygen (O_2_) mass transfer modeling (Sections 3.4, 2.11, and 2.12). This simulation pipeline provides a quantitative framework linking experimentally observed biomass organization, light transport dynamics, and metabolic processes. Importantly, the results suggest that biomass growth and clustering can reshape the internal light field and hence O_2_ distribution (due to photosynthesis), creating feedback mechanisms that may influence subsequent algal growth rates.

### 3.3 Respirometry measurements

Light-dependent O_2_ dynamics within the bioprinted algal constructs were quantified using respirometry by monitoring changes in dissolved O_2_ concentration in the bulk medium surrounding each construct during controlled illumination in a custom-built gas-exchange chamber (Murthy et al., 2025) (see Section 2.8). This approach provides a direct experimental measure of net and gross photosynthetic activity, thereby enabling quantitative assessment of construct-level metabolic performance.

**FIGURE 4:**
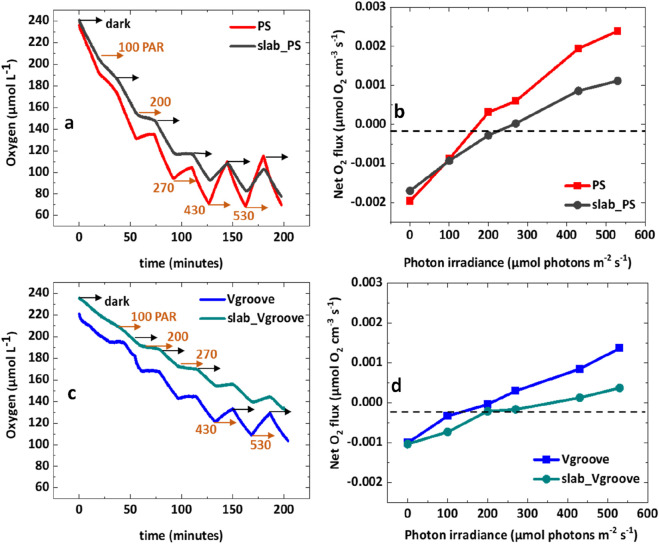
Respirometry-based assessment of light-dependent O_2_ exchange in bioprinted algal constructs under different light levels (PAR) with alternating dark measurements of **(a)** Representative time-resolved changes in O_2_ concentration, as measured under stepwise increases in photon irradiance (PAR; 400–700 nm) with alternating dark intervals for the perforated slab (PS) and its corresponding flat slab control (slab_PS); **(b)** Net oxygen production rates normalized to construct volume for PS and slab_PS, calculated from the linear slopes of the oxygen concentration traces shown in **(a)**. **(c)** Corresponding dissolved O_2_ concentration profiles for the V-groove construct and its flat slab control (slab_V-groove) measured under identical illumination conditions. **(d)** Volume-normalized net O_2_ production rates for the V-groove and slab_V-groove constructs derived from the slopes shown in **(c)**. All measurements were performed on day 4 after bioprinting (n = 1). Net O_2_ production rates were used to calculate net quantum efficiency and to parameterize O_2_ mass transfer simulations.

Figures 4a,c show representative time-resolved changes in dissolved O_2_ concentration, as measured during stepwise increases in photon irradiance with intervening dark periods, for the PS and V-groove geometries, respectively, together with their corresponding flat slab controls, on day 4. As photon irradiance increased, the structured constructs exhibited steeper positive slopes in dissolved O_2_ concentration after onset of light, indicating higher net O_2_ production rates compared to flat slabs under the tested conditions. This behavior is consistent with increased photosynthetic activity associated with differences in internal light availability and mass transfer within the structured geometries.

Net photosynthetic O_2_ production rates were calculated from the linear slopes of the O_2_ concentration vs. time recordings (Figures 4b,d). Net O_2_ production rates were normalized to construct volume to enable direct comparison between each structured geometry and its corresponding flat control, which had identical volumes. Both structured and flat constructs exhibited positive net O_2_ production above a photon irradiance of approximately 300 μmol photons m^-2^ s^-1^, consistent with the light saturation behavior observed in the variable chlorophyll fluorescence measurements (Figure 2). However, the PS and V-groove constructs showed higher net O_2_ production rates than their respective flat controls, suggesting higher construct-level photosynthetic output under these conditions.

Gross photosynthetic rates were estimated by combining the measured net O_2_ production rates with post-illumination dark respiration rates at each irradiance level (Supplementary Figure S10). Based on these measurements, the data point obtained at 430 μmol photons m^-2^ s^-1^, where all constructs exhibited robust net O_2_ production, was selected to calculate the net quantum efficiency (net-QE) of photosynthetic O_2_ evolution (Section 2.10). The resulting net-QE values for all samples on day 4 are summarized in Supplementary Table S2.

These experimentally derived net-QE values were subsequently used as quantitative inputs for O_2_ mass transfer simulations (Section 2.12), providing a quantitative link between measured metabolic behavior and predictive modeling. This workflow grounds the simulations in experimentally measured photosynthetic parameters rather than assumed values, thereby supporting the mechanistic interpretation of O_2_ transport and metabolic limitations within the bioprinted constructs.

### 3.4 Light and mass transfer simulation

Simulations of steady-state scalar irradiance and O_2_ transport were performed to analyze the physical factors that may contribute to the experimentally observed growth differences between structured and flat constructs. By integrating experimentally derived optical and metabolic parameters, these simulations offer a quantitative framework linking construct geometry, internal light distribution, O_2_ dynamics, and algal growth trends.

#### 3.4.1 PS versus slab_PS

**FIGURE 5:**
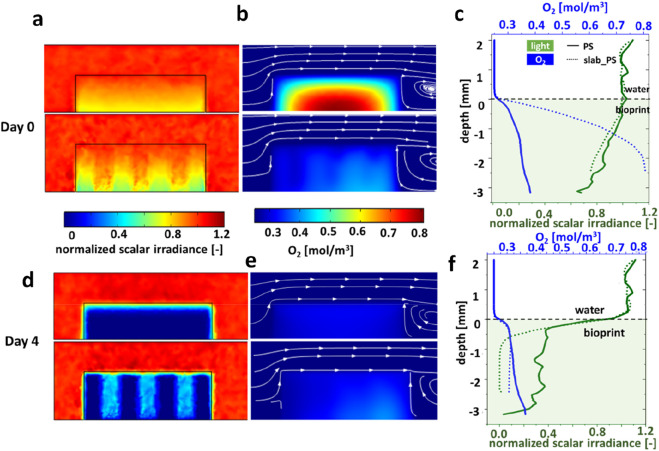
Simulated steady-state light and O_2_ distributions in bioprinted perforated slab (PS) and corresponding flat slab control (slab_PS) constructs. **(a)** Simulated vertical distribution of normalized scalar irradiance (λ = 636 nm) shown in a 2D cross-section of the constructs on day 0 (cross-section along the center of the geometry); **(b)** Corresponding simulated steady-state O_2_ concentration distribution on day 0; **(c)** One-dimensional depth profiles of normalized scalar irradiance (relative to incident irradiance) and O_2_ concentration extracted along the vertical line in the center of the geometry for day 0; **(d)** Simulated vertical distribution of normalized scalar irradiance (λ = 636 nm) in a 2D cross-section on day 4; **(e)** Corresponding simulated steady-state O_2_ concentration distribution on day 4. **(f)** One-dimensional depth profiles of normalized scalar irradiance and O_2_ concentration extracted along the same vertical line on day 4.

On day 0, the simulated 2D cross-sections of the internal light field (Figure 5a; Supplementary Figure S11a) and corresponding 1D depth profiles (Figure 5c; Supplementary Figure S11c) indicate that light penetrated through the full thickness of both PS and slab_PS constructs. The average simulated scalar irradiance was similar for PS and slab_PS (PS ≈ 95% of slab_PS; Supplementary Figure S6a). However, due to its perforated architecture, PS exhibited a 2.5-fold larger illuminated surface area compared to slab_PS, resulting in a more spatially distributed light field.

The corresponding simulated steady-state O_2_ distributions on day 0 (Figures 5b,c; Supplementary Figure S11b,c) revealed higher O_2_ accumulation within slab_PS than PS. This difference is attributed to more efficient O_2_ exchange between PS and the surrounding medium, enabled by its higher surface-area-to-volume ratio (2.5 times higher). As a result, the average steady-state O_2_ concentration in PS was approximately 65% of that in slab_PS on day 0 (Supplementary Figure S6a), despite similar overall light availability.

By day 4, biomass accumulation substantially altered the internal optical and O_2_ mass transfer conditions. Simulated light distributions (Figure 5d; Supplementary Figure S11a) and corresponding depth profiles (Figure 5f; Supplementary Figure S11c) show that in slab_PS, light was almost completely attenuated within the upper ∼500 μm of the construct. In contrast, PS maintained deeper light penetration, particularly in regions adjacent to the water channels, where light reached nearly the full construct depth. Regions farther from the channels still exhibited strong attenuation within the top ∼500 μm (Supplementary Figures S5d,S11a). Despite these spatial differences, the average simulated scalar irradiance in PS on day 4 remained comparable to that of slab_PS (∼96%; Supplementary Figure S6a).

Simulated steady-state O_2_ distributions on day 4 (Figures 5e,f; Supplementary Figures S11b,c) showed slightly higher O_2_ concentrations in deeper regions of PS relative to slab_PS. This behavior is consistent with the combination of deeper light penetration and higher experimentally measured net O_2_ production rates in PS (Section 3.3; Supplementary Table S2). Notably, although PS exhibited a 2.2-fold higher net O_2_ production rate (Figures 4a,b; Supplementary Figure S6a), its average steady-state O_2_ concentration was only marginally higher than that of slab_PS (PS ≈ 105% of slab_PS), reflecting the dominant role of enhanced mass transfer in removing O_2_ from the construct, owing 2.4 times higher surface area to volume ratio in PS, as compared to the corresponding slab.

Taken together, these results suggest that the PS geometry may sustain comparatively favorable growth conditions through the combined effects of enhanced light accessibility and efficient mass transfer. The increased illuminated surface area may help maintain sufficient internal light levels, which can support autotrophic photosynthesis throughout cultivation, even as biomass accumulates. The enhanced O_2_ flux simulated for PS reflects improved mass transfer within the construct and can be used as a proxy for the transport of other key chemical species, such as CO_2_ and dissolved nutrients, that are required to sustain photosynthesis and biomass growth. This improved exchange with the surrounding medium may mitigate transport limitations and could facilitate supplementary heterotrophic metabolism under locally light-limited conditions. Such dual metabolic contributions have been shown to support higher cell densities and growth rates (Barros et al., 2019; Doucha and Lívanský, 2012). Accordingly, the PS architecture may create conditions that are consistent with contributions from both autotrophic and heterotrophic processes, to promote higher biomass accumulation as observed under present experimental conditions.

#### 3.4.2 Vgroove versus slab_Vgroove

**FIGURE 6:**
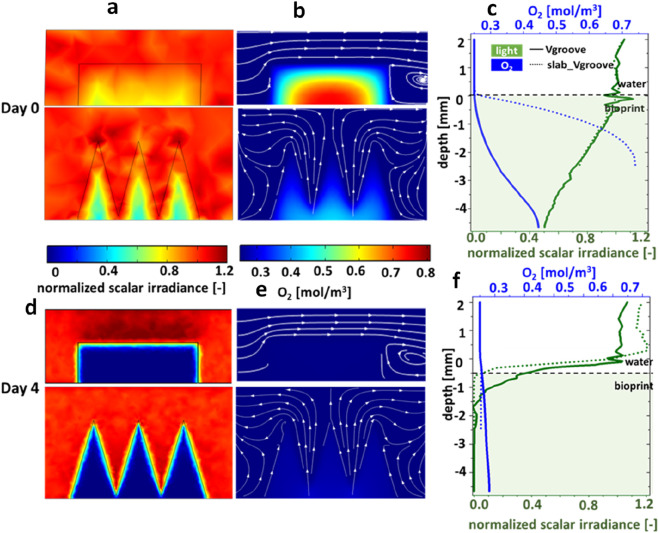
Simulated steady-state light and oxygen distributions in bioprinted V-groove and corresponding flat slab control (slab_V-groove) constructs. **(a)** Simulated vertical distribution of normalized scalar irradiance (λ = 636 nm) shown in a 2D cross-section, along the middle of the constructs, on day 0; **(b)** Corresponding simulated steady-state O_2_ concentration distribution on day 0; **(c)** One-dimensional depth profiles of normalized scalar irradiance (relative to incident irradiance) and O_2_ concentration extracted along the vertical line in the center of the construct, for day 0; **(d)** Simulated vertical distribution of normalized scalar irradiance (λ = 636 nm) in a 2D cross-section on day 4; **(e)** Corresponding simulated steady-state O_2_ concentration distribution on day 4; **(f)** One-dimensional depth profiles of normalized scalar irradiance and O_2_ concentration extracted along the same vertical line on day 4.

On day 0, simulations of the internal light field show that photon penetration extended throughout the entire V-groove and slab constructs, as indicated by the 2D cross-sections (Figure 6a) and corresponding 1D depth profiles (Figure 6c; Supplementary Figure S12). The average simulated scalar irradiance in the V-groove was comparable to that of the flat slab control (approximately 94% of slab_V-groove; Supplementary Figure S6b). However, the V-groove geometry provided a twofold increase in illuminated surface area relative to slab_V-groove, resulting in a more distributed internal light field.

Simulated steady-state O_2_ distributions on day 0 (Figures 6b,c; Supplementary Figure S12) revealed higher O_2_ accumulation within slab_V-groove than in the V-groove construct. This behavior is attributable to more efficient O_2_ exchange between the V-groove and the surrounding medium, enabled by its two-fold higher surface-area-to-volume ratio. Consequently, the average simulated steady-state O_2_ concentration in the V-groove on day 0 was approximately 70% of that in slab_V-groove (Supplementary Figure S6b), despite similar overall light availability.

By day 4, biomass accumulation substantially altered internal light and O_2_ mass transfer conditions. Simulated light distributions (Figure 6d) and corresponding depth profiles (Figure 6f; Supplementary Figure S12) indicate that in slab_V-groove, light was almost completely attenuated within the upper ∼500 µm of the construct. In contrast, the V-groove geometry maintained deeper light penetration, reaching approximately 1.5 mm near the groove peaks and ∼1 mm along the slanted surfaces, where increased reflective losses occurred due to the angled geometry. As a result, the average simulated scalar irradiance within the V-groove on day 4 was approximately 40% higher than that of slab_V-groove (Supplementary Figure S6b).

The simulated steady-state O_2_ distributions on day 4 (Figures 6e,f; Supplementary Figure S12) showed elevated O_2_ concentrations in deeper regions of the V-groove compared to slab_V-groove, which is consistent with increased local light availability and higher experimentally measured net O_2_ production rates. Although the V-groove exhibited an approximately ten-fold increase in net O_2_ production relative to its slab control (Figures 4c,d; Supplementary Figure S6b), the average simulated steady-state O_2_ concentration remained comparable between the two geometries (V-groove ≈ 106% of slab_V-groove). This outcome reflects the dominant role of enhanced mass transfer in facilitating O_2_ removal from the V-groove construct, thereby preventing excessive accumulation despite elevated photosynthetic activity.

Together, these results suggest that the moderate (1.3-fold) increase in algal growth observed for the V-groove geometry may arise from a balanced interplay between light availability and mass transfer, rather than from a single dominant mechanism. In contrast to the PS geometry, the growth advantage in the V-groove appears to be associated primarily with increased autotrophic activity enabled by improved light penetration relative to the flat slab, particularly as biomass accumulated over time. Simulations showed that by day 4, the V-groove maintained a ∼40% higher internal light availability than slab_V-groove (Supplementary Figure S6), while also exhibiting enhanced O_2_ exchange with the surrounding medium. These conditions may support higher photosynthetic activity within the construct and could also stimulate complementary heterotrophic metabolism under locally light-limited regions, contributing to biomass accumulation. Compared to PS, the lower surface-area-to-volume ratio of the V-groove (PS: 2.4; V-groove: 2, Supplementary Figure S6), may limit the extent of mass transfer enhancement, which is consistent with the more modest biomass increase observed relative to flat controls under the tested conditions.

Finally, we note that construct geometry can also influence local flow patterns around the bioprints, thereby affecting mass transfer. While the simulations presented here were designed to provide mechanistic insight and guide interpretation of experimental observations, they do not explicitly capture the full hydrodynamic complexity present during incubation and gas-exchange measurements. Experiments were conducted under stirred conditions, whereas the simulations assume simplified laminar flow. Future work will address these limitations by incorporating more realistic flow dynamics coupled to photosynthetic activity and transport processes.

## 4 Conclusions and outlook

We present a proof-of-concept study demonstrating a computation-driven, closed-loop framework that integrates predictive modeling, 3D bioprinting, quantitative experimentation, and post-experimental simulations to design and evaluate structured microalgal constructs. In this approach, mechanistic simulations of light transport and mass transfer are used to guide construct design, while experimental measurements, including photosynthetic performance, oxygen exchange, and biomass concentration, are subsequently incorporated into simulations to interpret observed growth trends and refine model predictions. This bidirectional coupling between computation and experiment provides mechanistic insight beyond empirical observation and minimizes reliance on trial-and-error design.

Using this framework, we observed that structured bioprinted geometries exhibited higher measured growth rates, under the present experimental conditions, compared to flat bioprinted slabs representative of simple biofilms. The higher biomass observed in these architectures may arise from synergistic improvements in light accessibility and mass transfer of chemical species. Increased illuminated surface area and more efficient solute exchange with the surrounding medium may support autotrophic photosynthesis as biomass accumulated, while also permitting complementary heterotrophic metabolism under locally light-limited conditions. Differences in surface-area-to-volume ratio and internal light redistribution are consistent with the distinct growth trends observed between PS and V-groove geometries.

Our study provides a pipeline for the systematic investigation and iterative refinement of phototrophic systems. Structured bioprinted constructs represent a potential route toward compact, high-density algal cultivation platforms for applications such as photobioreactors, addressing key limitations of conventional systems that rely on large land areas and inefficient light utilization (Suali and Sarbatly, 2012; Amer et al., 2011). By explicitly linking construct geometry to light management, mass transfer, and metabolic performance, the presented framework provides a predictive basis for optimizing space-efficient bioproduction systems for biofuels and bioproducts. Future studies will incorporate biological replication to rigorously evaluate the proposed design principles and to assess their reproducibility, performance implications, and growth trends in phototrophic systems.

The fabrication–measurement–simulation pipeline demonstrated here is broadly applicable to investigating structure–function relationships in other phototrophic and photosymbiotic systems, including reef-building corals (Brodersen et al., 2014; Murthy et al., 2023; Roth, 2014; Hatcher, 1988), jelly fish (Ames et al., 2020), and stratified microbial communities. Replicating and interrogating complex light–mass transfer interactions in such systems may yield insight into natural energy optimization strategies and ecological adaptations. Moreover, the platform holds potential for bio-inspired and synthetic biology applications, enabling the design of multi-cellular, spatially organized constructs to study symbiosis, microbe–host interactions, and metabolic interdependencies. These capabilities may also inform therapeutic design, targeted drug delivery, and tissue engineering applications. Future work will focus on expanding this platform to include multilayered 3D bioprints, e.g., inspired by the hierarchical organization of reef-building corals (Wangpraseurt et al., 2020) or complementary spectral absorption in stratified microbial biofilms (Kühl et al., 2020; Moßhammer et al., 2024), enabling spatial separation and canopy formations of different cell types with complementary functions. Additionally, we aim to incorporate more realistic hydrodynamic conditions, including turbulent and oscillatory flows commonly found in natural environments, to better replicate *in vivo* performance. Incorporating adaptive or responsive materials could further allow dynamic modulation of mass and light transfer, ultimately enabling the development of highly optimized, self-regulating bioreactors.

## Data Availability

The original contributions presented in the study are included in the article/Supplementary Material, further inquiries can be directed to the corresponding author.
