# Supplementary material for: Structured microalgal bioprints for enhanced photosynthetic performance and growth
Source: Front Bioeng Biotechnol. 2026 Jul 6;14:1755740. doi: 10.3389/fbioe.2026.1755740 (PMC13381435; doi:10.3389/fbioe.2026.1755740)
Supplement: Supplementary file 1 [file DataSheet1.pdf]

Structured Microalgal Bioprints for Enhanced Photosynthetic Performance and Growth

Swathi Murthy[1*], Maria Mosshammer[1], and Michael Kühl[1]

[1] Marine Biological Section, Department of Biology, University of Copenhagen,
Strandpromenaden 5, DK 3000 Helsingør, Denmark

[*]Corresponding author, email: swathi.murthy@bio.ku.dk

KEYWORDS: Predictive modeling, light transfer, mass transfer, bioprinting

Supplementary information

Table T1: Estimated optical properties of the samples on Day 4, as determined from the OCT images and cell counts. The last row in the table lists the optical properties on Day 0. PS: perforated slab; slab_PS: flat slab control corresponding to the PS geometry; slab_V-groove: flat slab control corresponding to the V-groove geometry. (n=1)

Sample	Cell numbers Day 4 X 10^6	Volume (mm^3)	Algal density (kgm^{-3})	μ_a (mm^{-1})	g (mm^{-1}) Based on OCT	μ_s (mm^{-1}) Based on OCT
PS	223	175.64	49.5	8.9	0.98	300
slab_PS	92	175.64	20.4	3.67	0.99	200
Vgroove	153	123.28	48.4	8.7	0.7	8
Slab_vgroove	117	123.28	37	6.6	0.9	100
Day 0	19	1000	0.75	0.135	0.99	2.25

Table T2: Model parameters for flow, mass transport and biokinetics. *PS: perforated slab; slab_PS: flat slab control corresponding to the PS geometry; slab_V-groove: flat slab control corresponding to the V-groove geometry.*

Parameter	Symbol	Value	Unit	Source
		Flow		
Viscosity water	η_{wat}	0.0009	Pa s	ref[65]
		Densities		
water	ρ_{wat}	997	kg m^{-3}	ref[65]
bio-print	ρ_{bp}	1109	kg m^{-3}	ref[66]
		Mass transport		
		Diffusion coefficients		
O_2 in water	$D_{O2,wat}$	$2{\cdot}10^{-9}$	m^2 s^{-1}	ref[67]
O_2 in bio-print	$D_{O2,bp}$	$1{\cdot}10^{-9}$	m^2 s^{-1}	ref[68]
		**Experimental parameters**		
Average water velocity	v_0	5	mm s^{-1}	ref[14]
Ambient temperature	T_0	25	°C	ref[14]
Inlet O_2 concentration	$c_{0,O2}$	0.25	mol m^{-3}	ref[69]
Incident downwelling photon irradiance (400 – 700 nm)	I_0	430	µmol photons m^{-2} s^{-1}	
Net-quantum efficiency_Day0; all samples	*net-QE_day0*	$5{\cdot}10^{-3}$	-	
Net-quantum efficiency_Day4_PS	*net-QE_PS*	$5{\cdot}10^{-4}$	-	Respirometry measurements
Net-quantum efficiency_Day4_slab_PS	*net-QE_slab_PS*	$5.4{\cdot}10^{-4}$	-	Respirometry measurements

Net-quantum efficiency_Day4_Vgroove	*net-QE_Vgroove*	$2.2 \cdot 10^{-4}$	-	Respirometry measurements
Net-quantum efficiency_Day4_slab_Vgroove	*net-QE_slab_Vgroove*	$4.4 \cdot 10^{-5}$	-	Respirometry measurements

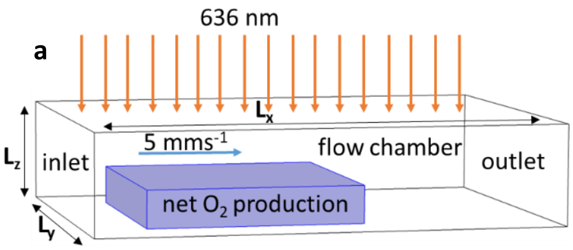

Figure S1: Schematic illustration of the model geometry for a slab in a flow chamber used for light, flow and mass transfer simulation. Downwelling irradiance at 636 nm illuminates the entire top boundary of the flow chamber. No light flux condition to all other boundaries. Simulations assumed a fully developed laminar flow with an average velocity of 5 mm s^{-1} and a constant O_2 concentration at the inlet. A fixed gauge pressure of zero and convection only condition was assumed at the outlet. We assumed a o slip boundary condition at the water bio-print interface, and symmetry and 'no flux' boundary conditions on the top and lateral walls of the flow chamber. A net photosynthetic O_2 production rate, calculated from the light field (MC simulation) within the biofilm, was assigned to the bioprint. L_x bioprint length + 12 mm, L_y bioprint width + 4mm, L_z bioprint height + 2 mm.

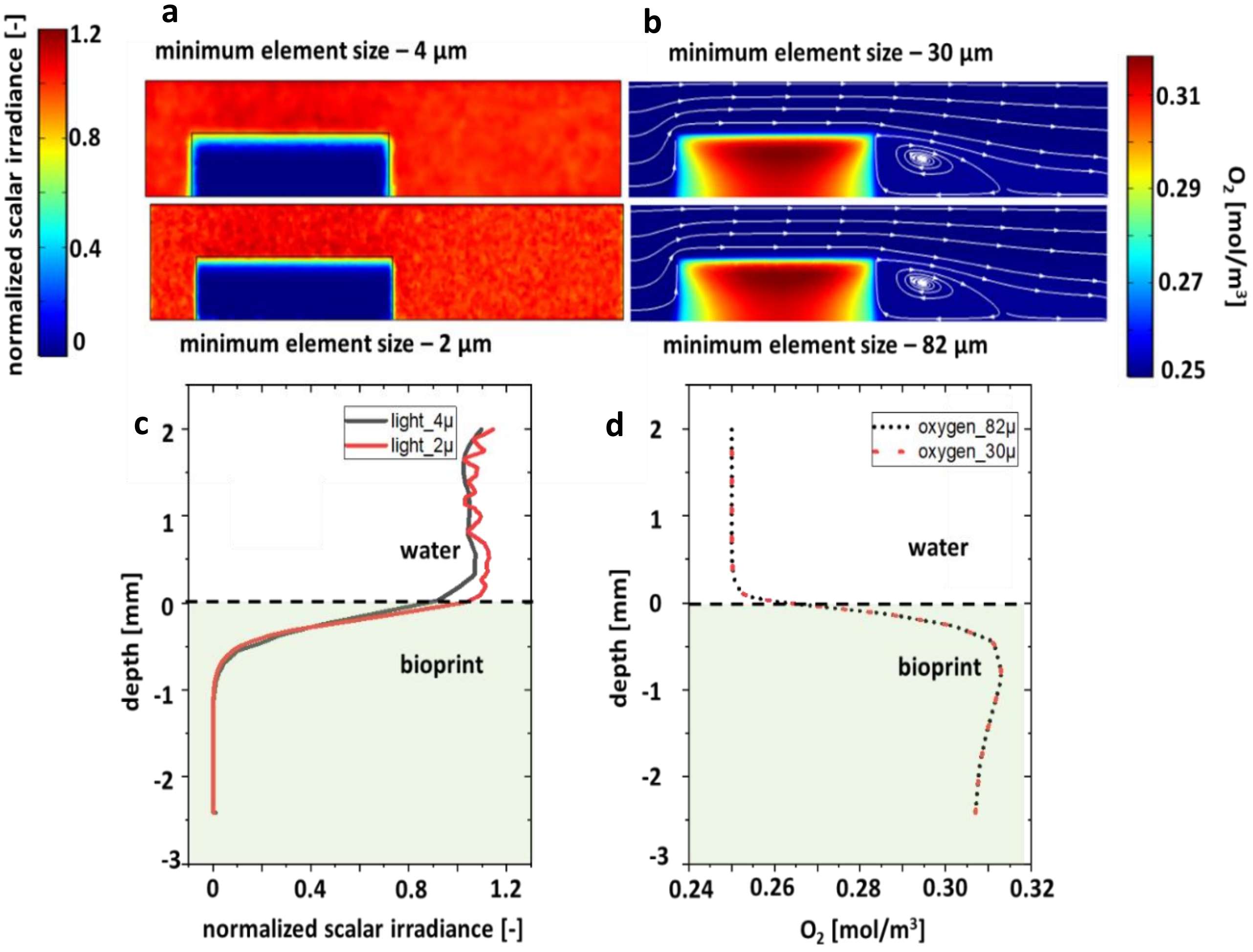

Figure S2: Mesh independent study for the slab_PS construct on day 4, ***a:*** *2D cross-sectional and* ***c:*** *1D plot of normalized scalar irradiance showing that the simulated light field (at 636 nm) is similar for meshes with minimum element size of 2 µm and 4 µm, respectively. A 4 µm minimum element size mesh was used for light simulations for the entire study.* ***b:*** *2D cross-sectional and* ***d:*** *1D plot showing that the simulated O_2 concentration (for downwelling irradiance of 430 µmol photons m^{-2} s^{-1} integrated over 400 – 700 nm) is identical for minimum element size of 30 µm and 82 µm. An 82µm minimum element size mesh was used for O_2 mass transfer simulation for the entire study.*

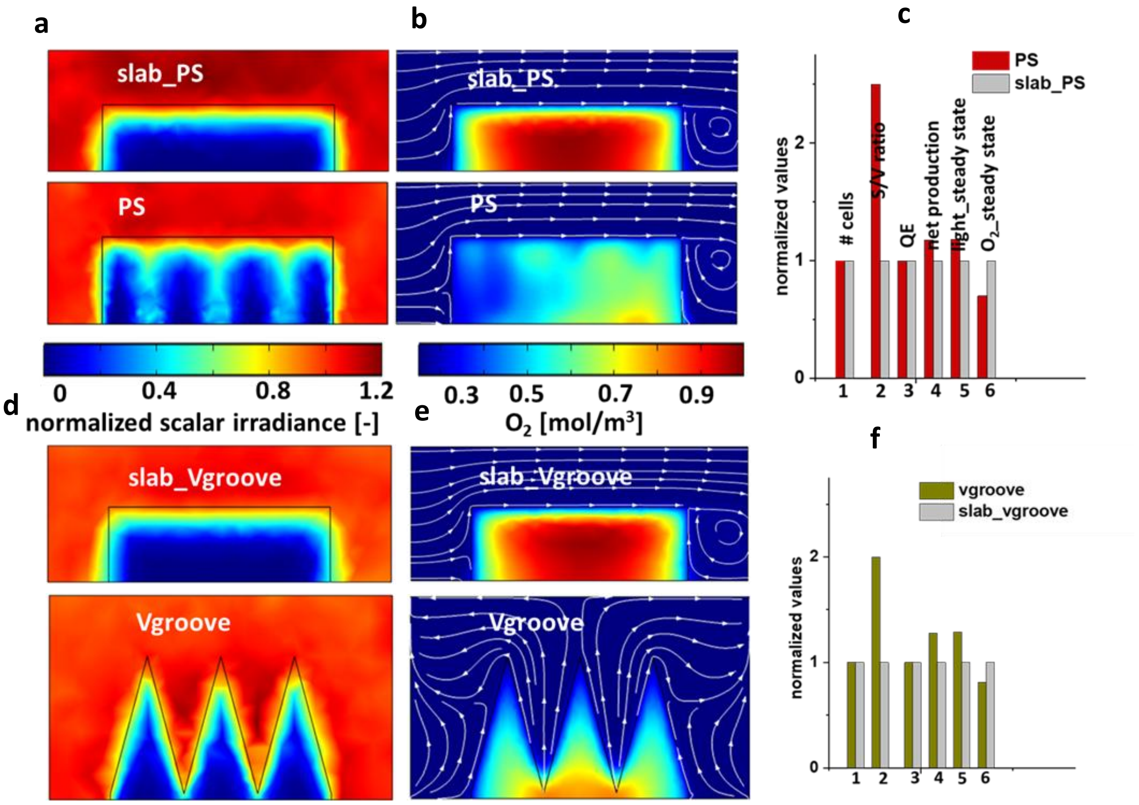

Figure S3: Steady state light and O_2 mass transfer simulations with assumed optical properties (μ_a = 1.7 mm^{-1}, μ_s = 90 mm^{-1}, g = 0.98, n = 1.33 at 636 nm) and a net photosynthetic quantum efficiency 0.005 at an incident photon irradiance of 320 µmol photons m^{-2} s^{-1} (400-700 nm). ***a:*** *2D cut plane data of light simulation of PS and slab_PS;* ***b:*** *2D cut plane data of corresponding O_2 mass transfer simulation for PS, slab_PS;* ***c:*** *Comparison between PS and slab_PS showing 17% higher light availability and 30% lesser oxygen accumulation for PS w.r.t slab_PS;* ***d:*** *2D cut plane data of light simulation of Vgroove and slab_Vgroove;* ***e:*** *2D cut plane data of corresponding O_2 mass transfer simulation for Vgroove, slab_Vgroove;* ***f:*** *Comparison between Vgroove and slab_Vgroove showing 27% higher light availability and 20% lesser oxygen accumulation for Vgroove vs. slab_Vgroove. PS: perforated slab; slab_PS: flat slab control corresponding to the PS geometry; slab_V-groove: flat slab control corresponding to the V-groove geometry.*

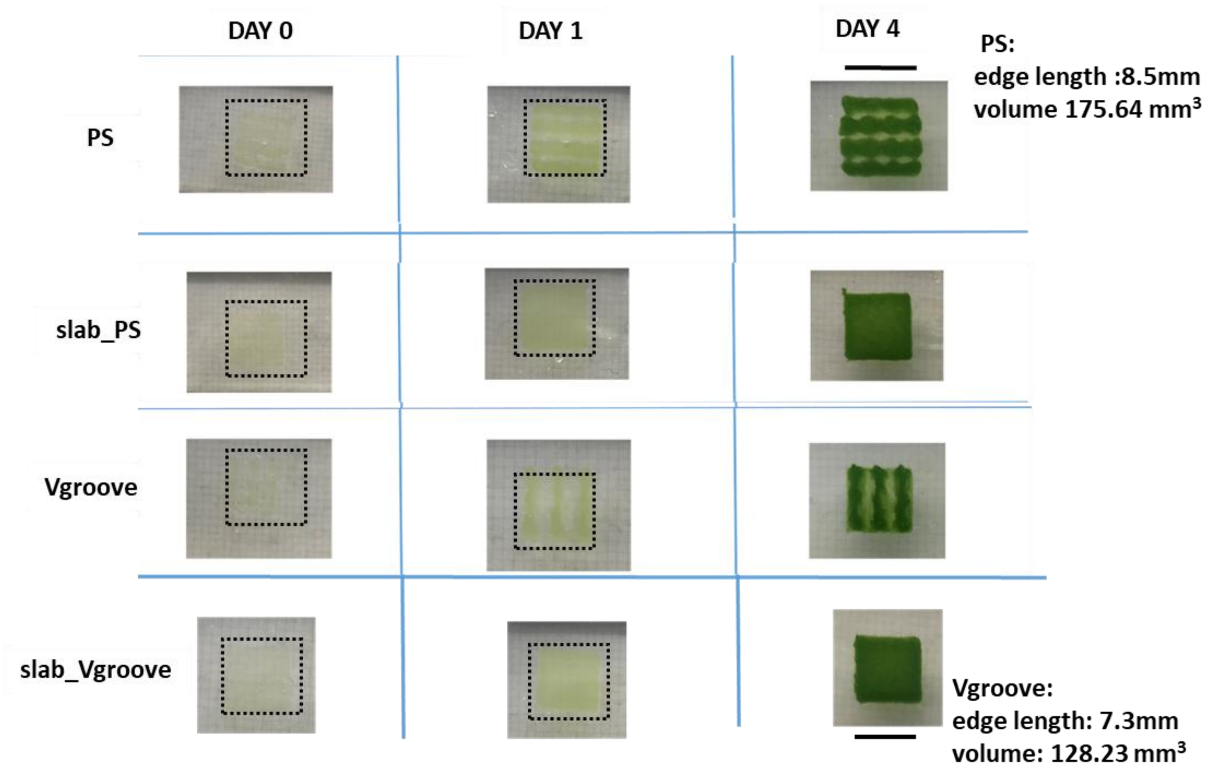

Figure S4: Photographs of bioprinted costructs, stored in TAP medium, taken on Day 0, Day 1 and Day 4. PS: perforated slab; slab_PS: flat slab control corresponding to the PS geometry; slab_V-groove: flat slab control corresponding to the V-groove geometry.

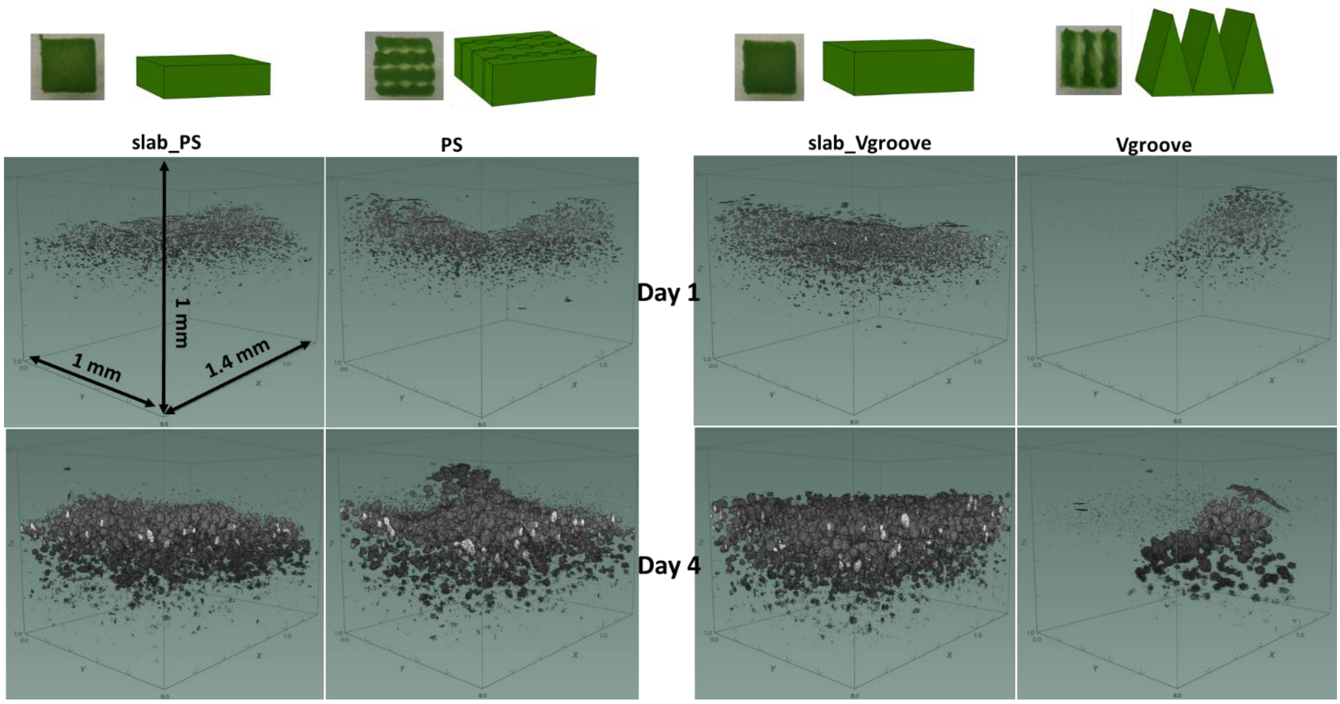

Figure S5: 3D rendering of optical coherence tomography (OCT) images of the different bioprinted geometries, shown in figure 4, on Day 1 and Day 4. See also the videos of slab_PS from Day 1 and Day 4, which show the aggregation of algal cells. PS: perforated slab; slab_PS: flat slab control corresponding to the PS geometry; slab_V-groove: flat slab control corresponding to the V-groove geometry.

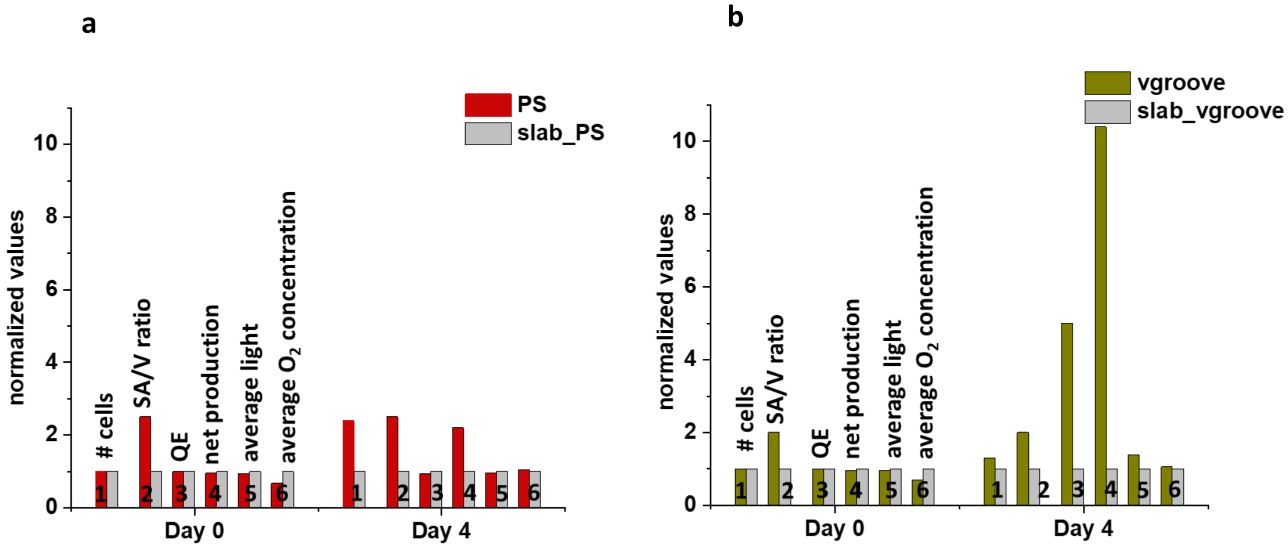

Figure S6: Comparison of various simulated parameters between the different bioprinted constructs and their respective slabs on Day 1 and Day 4, for ***a:*** *PS and slab_PS;* ***b:*** *Vgroove and slab_Vgroove. The light and O_2 values shown represent averages across the entire sample and are normalized to the corresponding slab, highlighting the differences between each 3D design and its slab counterpart. PS: perforated slab; slab_PS: flat slab control corresponding to the PS geometry; slab_V-groove: flat slab control corresponding to the V-groove geometry.*

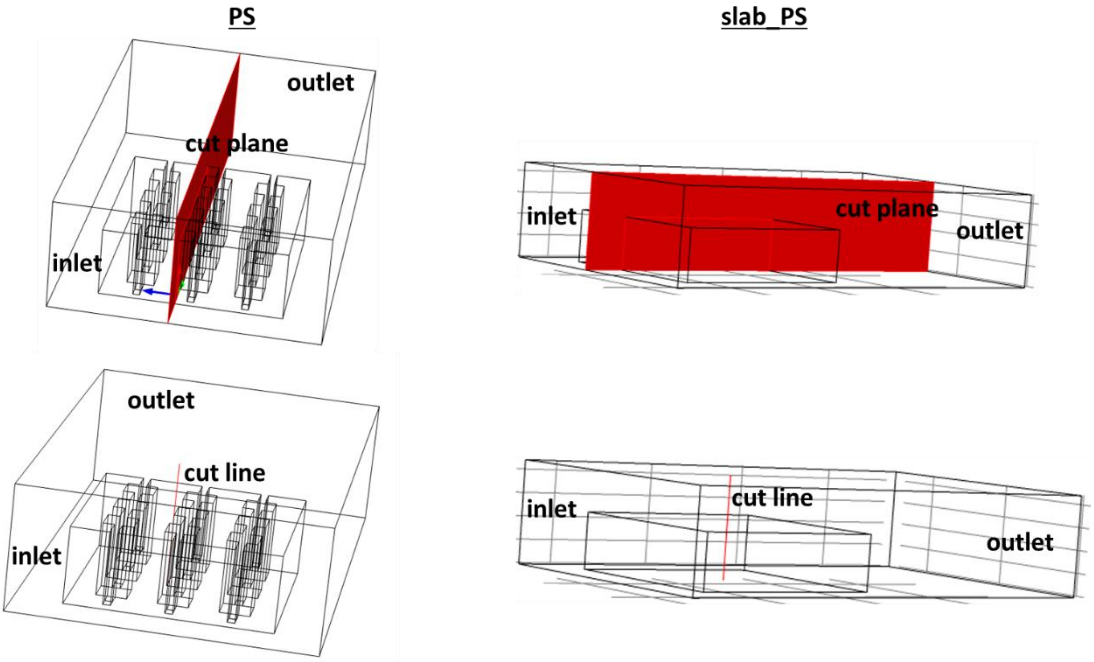

Figure S7: Schematic showing the position of cut plane and line for extraction of the simulation data for PS and slab_PS. PS: perforated slab; slab_PS: flat slab control corresponding to the PS geometry.

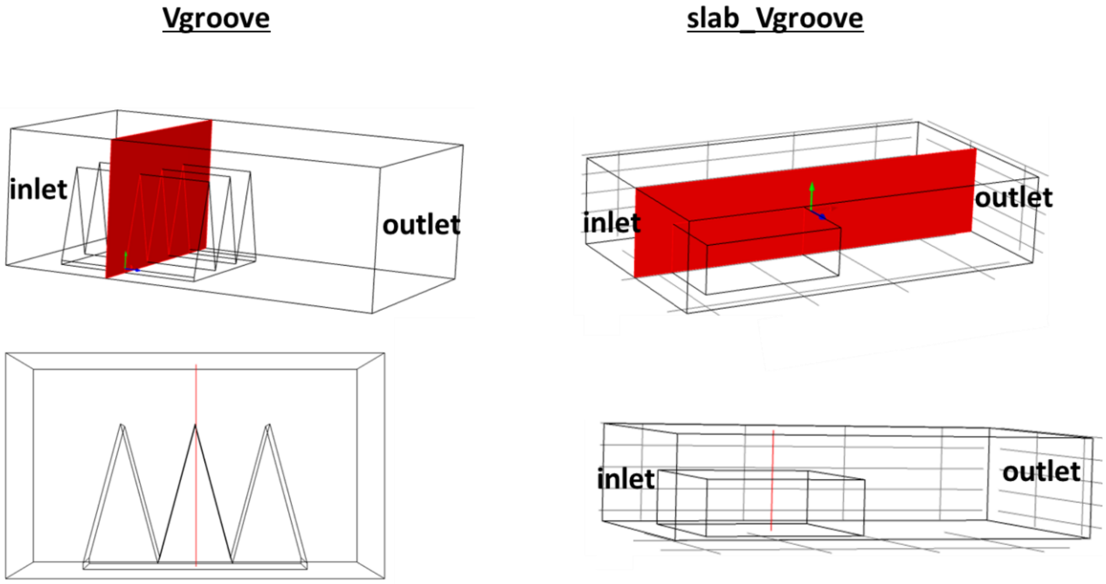

Figure S8: Schematic showing the position of cut plane and cut line for extraction of the simulation data for Vgroove and slab_Vgroove. slab_V-groove: flat slab control corresponding to the V-groove geometry.

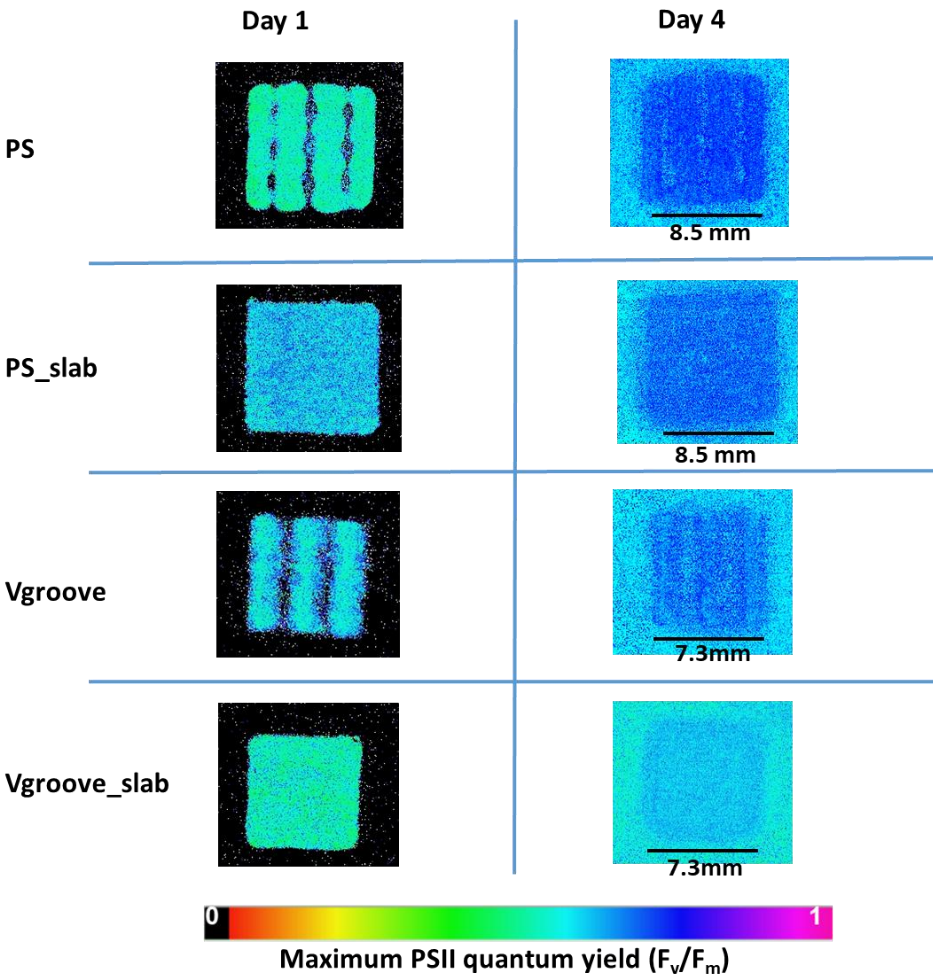

Figure S9: Maximum PSII quantum yield (F_v/F_m) images of all the samples on Day 1 and Day 4. PS: perforated slab; slab_PS: flat slab control corresponding to the PS geometry; slab_V-groove: flat slab control corresponding to the V-groove geometry.

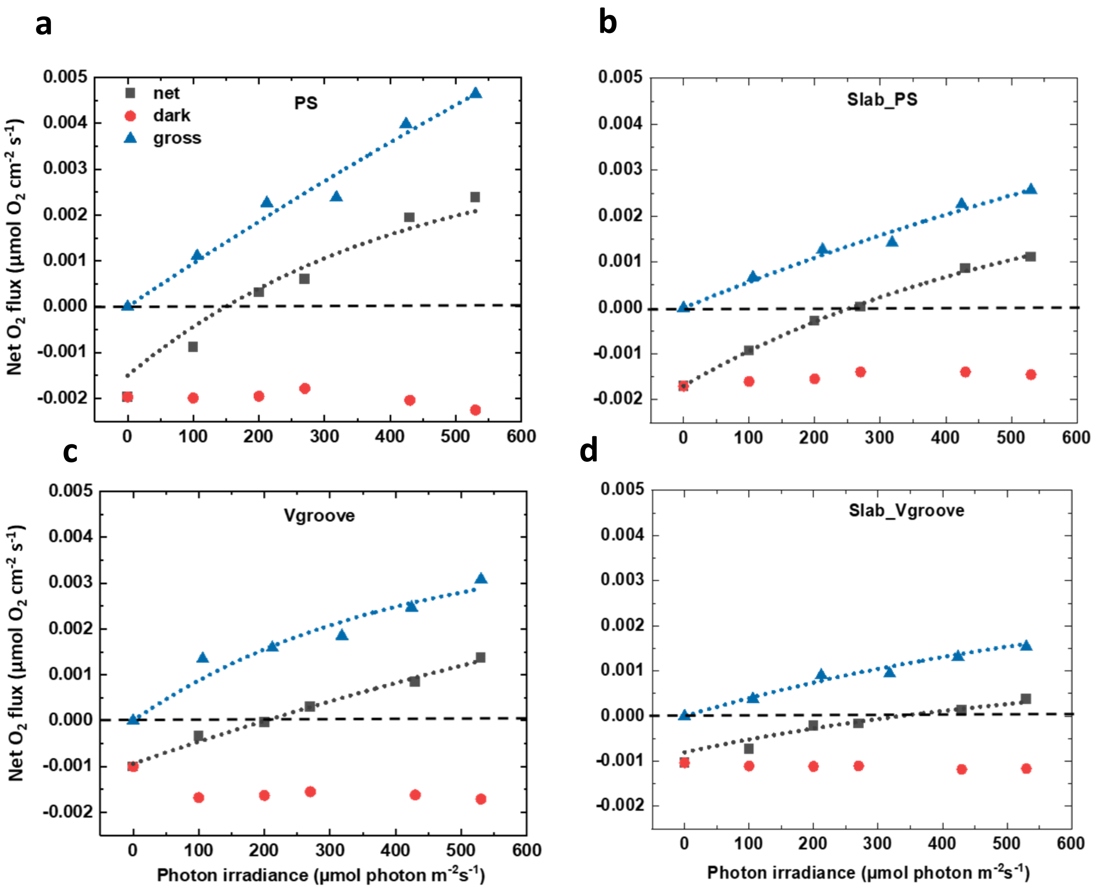

Figure S10: Net and estimated gross photosynthesis rates and dark respiration rates for different bioprinted construct geometries ***a:*** *PS;* ***b:*** *Slab_PS,* ***c:*** *Vgroove;* ***d:*** *Slab_Vgroove; as determined from the slopes in Figure 4a and 4c (symbols). The net production as a function of photon irradiance (400 – 700 nm) was fitted according to Spilling et al.*[1] *and the estimated gross production according to Webb et al.*[5] *(dotted lines). The rates are normalized to construct footprint area. PS: perforated slab; slab_PS: flat slab control corresponding to the PS geometry; slab_V-groove: flat slab control corresponding to the V-groove geometry.(n=1)*

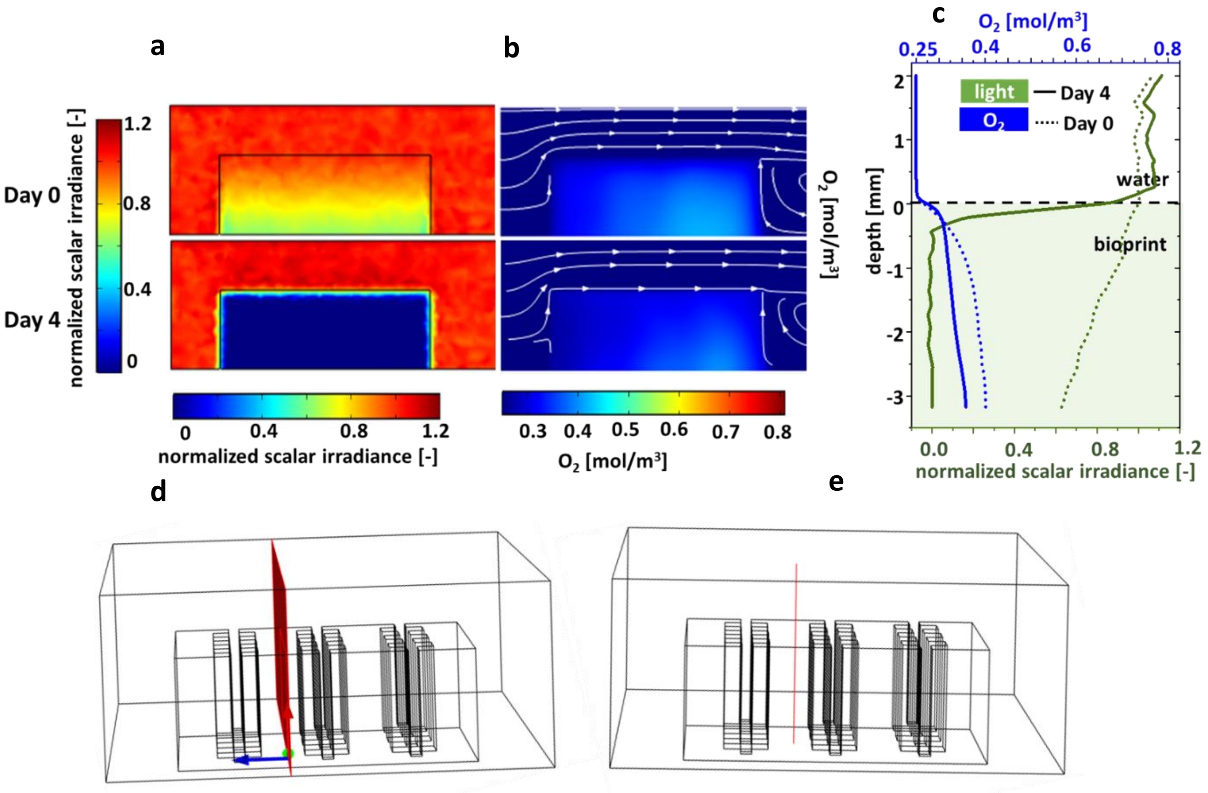

Figure S11: Steady state light and O_2 mass transfer simulation results for PS and slab_PS constructs along the middle plane. ***a:*** *2D cut plane data of light simulation on Day 0 and Day 4;* ***b:*** *2D cut plane data of corresponding O_2 mass transfer simulation on Day 0 and Day 4;* ***c:*** *Cut line data of both light and O_2 mass transfer simulations on Day 0 and Day 4.* ***d:*** *Schematic showing position of 2D cut plane;* ***e:*** *Schematic showing the position of cut line. PS: perforated slab; slab_PS: flat slab control corresponding to the PS geometry; slab_V-groove: flat slab control corresponding to the V-groove geometry.*

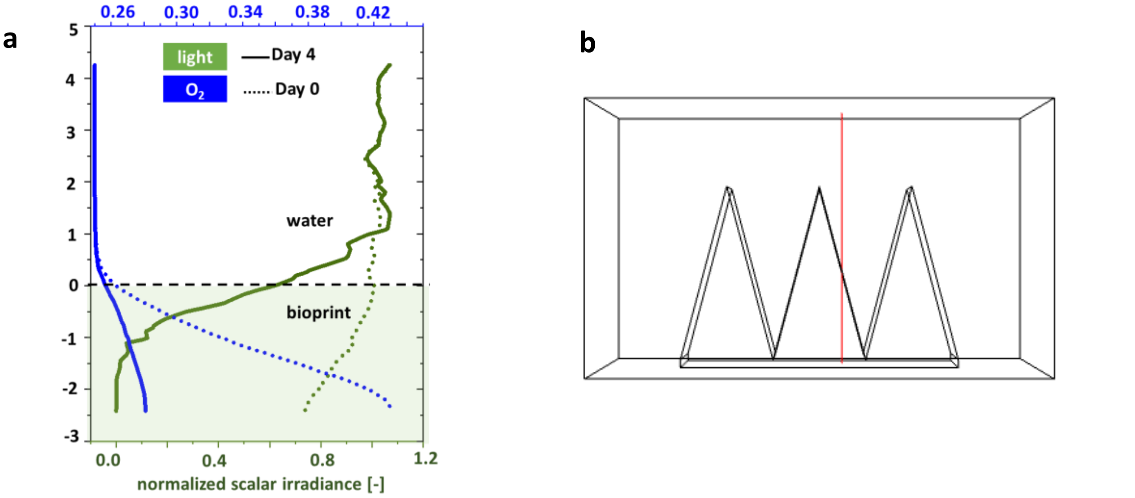

Figure S12: Steady state light and O_2 mass transfer simulation results for Vgroove. ***a:*** *Cut line data of both light and O_2 mass transfer simulations on Day 0 and Day 4.* ***b:*** *Schematic showing the position of the cut line. PS: perforated slab; slab_PS: flat slab control corresponding to the PS geometry; slab_V-groove: flat slab control corresponding to the V-groove geometry.*

References

1 Spilling, K., Titelman, J., Greve, T. M. & Kühl, M. MICROSENSOR MEASUREMENTS OF THE EXTERNAL AND INTERNAL MICROENVIRONMENT OF FUCUS VESICULOSUS (PHAEOPHYCEAE)1. *Journal of Phycology* **46**, 1350-1355, doi:10.1111/j.1529-8817.2010.00894.x (2010).

2 Suali, E. & Sarbatly, R. Conversion of microalgae to biofuel. *Renewable and Sustainable Energy Reviews* **16**, 4316-4342, doi:https://doi.org/10.1016/j.rser.2012.03.047 (2012).

3 Mata, T. M., Martins, A. A. & Caetano, N. S. Microalgae for biodiesel production and other applications: A review. *Renewable and Sustainable Energy Reviews* **14**, 217-232, doi:https://doi.org/10.1016/j.rser.2009.07.020 (2010).

4 Amer, L., Adhikari, B. & Pellegrino, J. Technoeconomic analysis of five microalgae-to-biofuels processes of varying complexity. *Bioresource Technology* **102**, 9350-9359, doi:https://doi.org/10.1016/j.biortech.2011.08.010 (2011).

5 Webb, W. L., Newton, M. & Starr, D. Carbon dioxide exchange of Alnus rubra. *Oecologia* **17**, 281-291, doi:10.1007/BF00345747 (1974).

6 Cho, C. *et al.* Study of Optical Configurations for Multiple Enhancement of Microalgal Biomass Production. *Scientific Reports* **9**, 1723, doi:10.1038/s41598-018-38118-w (2019).

7 Ruiz, J., Wijffels, R. H., Dominguez, M. & Barbosa, M. J. Heterotrophic vs autotrophic production of microalgae: Bringing some light into the everlasting cost controversy. *Algal Research* **64**, 102698, doi:https://doi.org/10.1016/j.algal.2022.102698 (2022).

8 Barros, A. *et al.* Heterotrophy as a tool to overcome the long and costly autotrophic scale-up process for large scale production of microalgae. *Scientific Reports* **9**, 13935, doi:10.1038/s41598-019-50206-z (2019).

9 Doucha, J. & Lívanský, K. Production of high-density Chlorella culture grown in fermenters. *Journal of Applied Phycology* **24**, 35-43, doi:10.1007/s10811-010-9643-2 (2012).

10 Bengtsson, M. M., Wagner, K., Schwab, C., Urich, T. & Battin, T. J. Light availability impacts structure and function of phototrophic stream biofilms across domains and trophic levels. *Molecular Ecology* **27**, 2913-2925, doi:10.1111/mec.14696 (2018).

11 Parmar, A., Singh, N. K., Pandey, A., Gnansounou, E. & Madamwar, D. Cyanobacteria and microalgae: A positive prospect for biofuels. *Bioresource Technology* **102**, 10163-10172, doi:https://doi.org/10.1016/j.biortech.2011.08.030 (2011).

12 Chua, S. T. *et al.* Light management by algal aggregates in living photosynthetic hydrogels. *Proc Natl Acad Sci U S A* **121**, e2316206121, doi:10.1073/pnas.2316206121 (2024).

13 Stambler, N. & Dubinsky, Z. Corals as light collectors: an integrating sphere approach. *Coral Reefs* **24**, 1-9, doi:10.1007/s00338-004-0452-4 (2005).

14 Brodersen, K. E., Lichtenberg, M., Ralph, P. J., Kühl, M. & Wangpraseurt, D. Radiative energy budget reveals high photosynthetic efficiency in symbiont-bearing corals. *Journal of The Royal Society Interface* **11**, 20130997, doi:doi:10.1098/rsif.2013.0997 (2014).

15 Holt, A. L., Rehm, L. F. & Sweeney, A. M. Simple Mechanism for Optimal Light-Use Efficiency of Photosynthesis Inspired by Giant Clams. *PRX Energy* **3**, 023014, doi:10.1103/PRXEnergy.3.023014 (2024).

16 Murthy, S., Mosshammer, M., Trampe, E. & Kühl, M. Functional imaging of 3D bioprinted microalgal constructs and simulation of their photosynthetic performance. *bioRxiv*, 2025.2002.2019.639067, doi:10.1101/2025.02.19.639067 (2025).

17 Sassaroli, A. & Martelli, F. Equivalence of four Monte Carlo methods for photon migration in turbid media. *J. Opt. Soc. Am. A* **29**, 2110-2117, doi:10.1364/JOSAA.29.002110 (2012).

18 Leino, A. A., Pulkkinen, A. & Tarvainen, T. ValoMC: a Monte Carlo software and MATLAB toolbox for simulating light transport in biological tissue. *Osa Continuum* **2**, 957-972, doi:10.1364/Osac.2.000957 (2019).

19 Zhu, C. & Liu, Q. Review of Monte Carlo modeling of light transport in tissues. *Journal of Biomedical Optics* **18**, 050902 (2013).

20 Yuan, Y., Yan, S. & Fang, Q. Light transport modeling in highly complex tissues using the implicit mesh-based Monte Carlo algorithm. *Biomed. Opt. Express* **12**, 147-161, doi:10.1364/BOE.411898 (2021).
21 Taylor Parkins, S. K., Murthy, S., Picioreanu, C. & Kühl, M. Multiphysics modelling of photon, mass and heat transfer in coral microenvironments. *Journal of The Royal Society Interface* **18**, 20210532, doi:10.1098/rsif.2021.0532 (2021).
22 Murthy, S., Picioreanu, C. & Kühl, M. Modeling the radiative, thermal and chemical microenvironment of 3D scanned corals. *Frontiers in Marine Science* **Volume 10 - 2023**, doi:10.3389/fmars.2023.1160208 (2023).
23 Levato, R. *et al.* From Shape to Function: The Next Step in Bioprinting. *Advanced Materials* **32**, 1906423, doi:10.1002/adma.201906423 (2020).
24 Groll, J. *et al.* Biofabrication: reappraising the definition of an evolving field. *Biofabrication* **8**, 013001, doi:10.1088/1758-5090/8/1/013001 (2016).
25 Hospodiuk, M., Dey, M., Sosnoski, D. & Ozbolat, I. T. The bioink: A comprehensive review on bioprintable materials. *Biotechnol Adv* **35**, 217-239, doi:10.1016/j.biotechadv.2016.12.006 (2017).
26 Derby, B. Printing and Prototyping of Tissues and Scaffolds. *Science* **338**, 921-926, doi:10.1126/science.1226340 (2012).
27 Zhang, T., Yan, K. C., Ouyang, L. & Sun, W. Mechanical characterization of bioprinted in vitro soft tissue models. *Biofabrication* **5**, 045010, doi:10.1088/1758-5082/5/4/045010 (2013).
28 Schuurman, W. *et al.* Gelatin-methacrylamide hydrogels as potential biomaterials for fabrication of tissue-engineered cartilage constructs. *Macromol Biosci* **13**, 551-561, doi:10.1002/mabi.201200471 (2013).
29 Billiet, T., Vandenhaute, M., Schelfhout, J., Van Vlierberghe, S. & Dubruel, P. A review of trends and limitations in hydrogel-rapid prototyping for tissue engineering. *Biomaterials* **33**, 6020-6041, doi:10.1016/j.biomaterials.2012.04.050 (2012).
30 Krujatz, F. *et al.* Green bioprinting: Viability and growth analysis of microalgae immobilized in 3D-plotted hydrogels versus suspension cultures. *Engineering in Life Sciences* **15**, 678-688, doi:https://doi.org/10.1002/elsc.201400131 (2015).
31 Wangpraseurt, D. *et al.* Bionic 3D printed corals. *Nature Communications* **11**, 1748, doi:10.1038/s41467-020-15486-4 (2020).
32 Tang, J. *et al.* Microalgae-Based 3D Bioprinting: Recent Advances, Applications and Perspectives. *Mar Drugs* **23**, doi:10.3390/md23090342 (2025).
33 Fanesi, A., Paule, A., Bernard, O., Briandet, R. & Lopes, F. The Architecture of Monospecific Microalgae Biofilms. *Microorganisms* **7**, 352 (2019).
34 Parker, A. R. & Townley, H. E. Biomimetics of photonic nanostructures. *Nature Nanotechnology* **2**, 347-353, doi:10.1038/nnano.2007.152 (2007).
35 Burresi, M. *et al.* Bright-White Beetle Scales Optimise Multiple Scattering of Light. *Scientific Reports* **4**, 6075, doi:10.1038/srep06075 (2014).
36 Dimijian, G. G. Evolving together: the biology of symbiosis, part 1. *Proc (Bayl Univ Med Cent)* **13**, 217-226 (2000).
37 Lupp, C. Host–microbe interactions. *Nature* **449**, 803-803, doi:10.1038/449803a (2007).
38 Medzhitov, R. Recognition of microorganisms and activation of the immune response. *Nature* **449**, 819-826, doi:10.1038/nature06246 (2007).
39 Turnbaugh, P. J. *et al.* The Human Microbiome Project. *Nature* **449**, 804-810, doi:10.1038/nature06244 (2007).
40 Loessner, D. *et al.* Functionalization, preparation and use of cell-laden gelatin methacryloyl–based hydrogels as modular tissue culture platforms. *Nature Protocols* **11**, 727-746, doi:10.1038/nprot.2016.037 (2016).

41 Liu, J., Hwang, H. H., Wang, P., Whang, G. & Chen, S. Direct 3D-printing of cell-laden constructs in microfluidic architectures. *Lab Chip* **16**, 1430-1438, doi:10.1039/c6lc00144k (2016).
42 Wangpraseurt, D., Wentzel, C., Jacques, S. L., Wagner, M. & Kühl, M. In vivo imaging of coral tissue and skeleton with optical coherence tomography. *Journal of The Royal Society Interface* **14** (2017).
43 Wangpraseurt, D. *et al.* Microscale light management and inherent optical properties of intact corals studied with optical coherence tomography. *bioRxiv*, 376723, doi:10.1101/376723 (2018).
44 Samatham, R. & Jacques, S. *Determine scattering coefficient and anisotropy of scattering of tissue phantoms using reflectance-mode confocal microscopy*. Vol. 7187 PWB (SPIE, 2009).
45 Samatham, R., Jacques, S. & Campagnola, P. Optical properties of mutant versus wild-type mouse skin measured by reflectance-mode confocal scanning laser microscopy (rCSLM). *Journal of Biomedical Optics* **13**, 041309 (2008).
46 Bao, D. *et al.* Automated detection and growth tracking of 3D bio-printed organoid clusters using optical coherence tomography with deep convolutional neural networks. *Frontiers in Bioengineering and Biotechnology* **Volume 11 - 2023**, doi:10.3389/fbioe.2023.1133090 (2023).
47 Tashman, J. W. *et al.* In situvolumetric imaging and analysis of FRESH 3D bioprinted constructs using optical coherence tomography. *Biofabrication* **15**, doi:10.1088/1758-5090/ac975e (2022).
48 Schreiber, U. in *Chlorophyll a Fluorescence: A Signature of Photosynthesis* (eds George Christos Papageorgiou & Govindjee) 279-319 (Springer Netherlands, 2004).
49 Ralph, P. J., Schreiber, U., Gademann, R., Kühl, M. & Larkum, A. W. D. CORAL PHOTOBIOLOGY STUDIED WITH A NEW IMAGING PULSE AMPLITUDE MODULATED FLUOROMETER1. *Journal of Phycology* **41**, 335-342, doi:10.1111/j.1529-8817.2005.04034.x (2005).
50 Schuurmans, R. M., van Alphen, P., Schuurmans, J. M., Matthijs, H. C. P. & Hellingwerf, K. J. Comparison of the Photosynthetic Yield of Cyanobacteria and Green Algae: Different Methods Give Different Answers. *PLOS ONE* **10**, e0139061, doi:10.1371/journal.pone.0139061 (2015).
51 Ralph, P. J. & Gademann, R. Rapid light curves: A powerful tool to assess photosynthetic activity. *Aquatic Botany* **82**, 222-237, doi:https://doi.org/10.1016/j.aquabot.2005.02.006 (2005).
52 Murthy, S., Mosshammer, M., Trampe, E. & Kühl, M. Functional imaging of 3D bioprinted microalgal constructs and simulation of their photosynthetic performance. *Biofabrication* **17**, 045010, doi:10.1088/1758-5090/adf9ca (2025).
53 Kandilian, R. *et al.* Simple method for measuring the spectral absorption cross-section of microalgae. *Chemical Engineering Science* **146**, 357-368, doi:https://doi.org/10.1016/j.ces.2016.02.039 (2016).
54 Al-Najjar, M. A. A., de Beer, D., Jørgensen, B. B., Kühl, M. & Polerecky, L. Conversion and conservation of light energy in a photosynthetic microbial mat ecosystem. *The ISME Journal* **4**, 440-449, doi:10.1038/ismej.2009.121 (2010).
55 Wang, L., Jacques, S. L. & Zheng, L. MCML--Monte Carlo modeling of light transport in multi-layered tissues. *Comput Methods Programs Biomed* **47**, 131-146, doi:10.1016/0169-2607(95)01640-f (1995).
56 Prahl, S., Keijzer, M., Jacques, S. & Welch, A. A Monte Carlo Model of Light Propagation in Tissue. *SPIE Inst. Ser. IS* **5** (1989).
57 Schreiber, U. Pulse-Amplitude-Modulation (PAM) Fluorometry and Saturation Pulse Method: An Overview. *Advances in Photosynthesis and Respiration* **19**, doi:10.1007/978-1-4020-3218-9_11 (2004).

58 Huang, D. *et al.* Optical coherence tomography. *Science* **254**, 1178-1181, doi:10.1126/science.1957169 (1991).
59 Levitz, D. *et al.* Quantitative characterization of developing collagen gels using optical coherence tomography. *J Biomed Opt* **15**, 026019, doi:10.1117/1.3377961 (2010).
60 Roth, M. S. The engine of the reef: photobiology of the coral–algal symbiosis. *Frontiers in Microbiology* **5**, doi:10.3389/fmicb.2014.00422 (2014).
61 Hatcher, B. G. Coral reef primary productivity: A beggar's banquet. *Trends in Ecology & Evolution* **3**, 106-111, doi:https://doi.org/10.1016/0169-5347(88)90117-6 (1988).
62 Ames, C. L. *et al.* Cassiosomes are stinging-cell structures in the mucus of the upside-down jellyfish Cassiopea xamachana. *Communications Biology* **3**, 67, doi:10.1038/s42003-020-0777-8 (2020).
63 Kühl, M. *et al.* Substantial near-infrared radiation-driven photosynthesis of chlorophyll f-containing cyanobacteria in a natural habitat. *Elife* **9**, doi:10.7554/eLife.50871 (2020).
64 Moßhammer, M., Trampe, E., Frigaard, N.-U. & Kühl, M. *Near infrared radiation-driven oxygenic photosynthesis contributes substantially to primary production in biofilms harboring chlorophyll f -containing cyanobacteria*. (2024).
65 *Fundamentals of heat and mass transfer*. (Sixth edition / [Frank P. Incropera ... [and others]. Hoboken, NJ : John Wiley, [2007] ©2007, 2007).
66 Hasgall PA, D. G. F., Baumgartner C, Neufeld E, Lloyd B, Gosselin MC, Payne D, Klingenböck A, Kuster N in

(2018).
67 CRC Handbook of Chemistry and Physics, 86th Edition Edited by David R. Lide (National Institute of Standards and Technology). CRC Press (an imprint of Taylor and Francis Group): Boca Raton, FL. 2005. 2544 pp. $125.96. ISBN 0-8493-0486-5. *Journal of the American Chemical Society* **128**, 5585-5585, doi:10.1021/ja059868l (2006).
68 MacDougall, J. D. & McCabe, M. Diffusion coefficient of oxygen through tissues. *Nature* **215**, 1173-1174, doi:10.1038/2151173a0 (1967).
69 Haas, A. F. *et al.* Visualization of oxygen distribution patterns caused by coral and algae. *PeerJ* **1**, e106-e106, doi:10.7717/peerj.106 (2013).
